# Multi-center chameleon hashing based Blockchain integrated digital copyright transaction scheme for data redacting in Blockchain based IoT systems

**DOI:** 10.1038/s41598-026-45111-1

**Published:** 2026-03-20

**Authors:** Lu Chen, Aniruddha Bhattacharjya, Yuwei Sun, Zixuan Wang, Zhixin Sun, Yichen Yu

**Affiliations:** 1 School of Communication and Artificial Intelligence, School of Integrated Circuits, Nanjing Institute of Technology, Nanjing, China; 2 Department of Electronic Engineering, Tsinghua University, Beijing, 100190 China; 3 BCBRBAB Intercontinental Trading Solutions Private Limited, New Delhi, India; 4 Engineering Research Center of Broadband Wireless Communication Technology of the Ministry of Education, Nanjing, China; 5 Nanjing University of Posts and Telecommunications, Nanjing, China; 6 University of North Carolina at Chapel Hill, Hinton James Residence Hall, Room 613A, 515 Hinton James Drive, Chapel Hill, NC 27514 USA; 7 Department of Electronic Engineering, Tsinghua University, Beijing, 100190 China

**Keywords:** Attribute encryption, Blockchain, Copyright protection, Copyright transaction, Node security management, Blockchain based Internet of Things (BIoT), Blockchain based Industrial Internet of Things (BIIOT), Engineering, Mathematics and computing

## Abstract

Traditional digital copyright protection relies on centralized authorities, which often lacks transparency and struggle to guarantee data security during ownership transfer. Furthermore, ownership changes require re-confirmation of copyrightwhich costs more time and wastes storage resources. Here a blockchain-based digital copyright transaction scheme is proposed which supports data redaction. Here multi-center chameleon hashing is used for modifying the copyright information in the transaction process. At the same time, a flexible and controllable permission node management mechanism is designed, proposed and implemented here and the optimized attribute encryption mechanism is used for providing the security protection for the node private key. The experimental results and security analysis have shown that the scheme has satisfied the data confidentiality of the copyright transferor, resistance to malicious nodes, and the confidentiality and accountability of the private key of the chameleon hash. In the scenario with more permission nodes, the private key broadcast in the node exit stage has higher efficiency and flexibility, so it’s effective in ensuring data confidentiality of the copyright transferor, resistance to malicious nodes, and the confidentiality and accountability of the private key of the chameleon hash in Blockchain based Internet of Things (BIoT) systems and it can be further used in Blockchain based Industrial Internet of Things (BIIOT) of present time.

## Introduction

Satoshi Nakamoto’s^1^ invention of the Blockchain network in 2008 has very deep impact in the present era. In present era, many diversified works and researches are going on the applicability of the Blockchain technology in the field of CPS systems^2–13^. In^8^ we have found that the 5 level architecture so called 5 C-CPS and it was foreknown for continuing CPSs in industrial sectors. We have seen in^13^ that three widely adopted distributed ledger platforms for IoT and CPS applications^14–17^ are Hyperledger Fabric^18^, IOTA^19,26^ and Ethereum^20,21^. In these studies^14–21^ the profound ways of using the Blockchain technology in Internet of Things (IoT) (characterized as BIoT) and CPS, were enlightened. Furthermore, recent research has extended blockchain applications to zero-trust environments and challenging terrains, demonstrating its versatility in securing IoT networks^22^.

We have three groupings of Consensus protocols for Blockchain^23–36^ in the present time, they are as follows- permissionless (Bitcoin and Ethereum) consensus, Semi-decentralized (Ripple along with Stellar are the examples) consensus, and consortium (BFT (Byzantine Fault Tolerance)) consensus protocol.

In the era of BIoT and BIIoT, the digitization of works facilitates the transaction and dissemination of digital content. These days, Infringement problems such as copying and modification of digital content are becoming more and more serious, causing damage to the interests of digital copyright owners. Therefore, there is an urgent need to protect digital copyright. In recent years, digital watermarking^36–38^, cryptography^39–43^, digital content retrieval^44^, big data and other technologies have been applied to copyright management, which has promoted the improvement of the digital copyright protection system to a certain extent. Copyright protection technology based on cryptography technology mainly achieves controllable authorization by protecting the secure distribution of digital content^45–47^. Copyright protection methods based on digital watermark technology are usually used to protect the digital content itself and can be used to protect the authorship of the digital content, control the integrity of the data, and verify the source of the data. It is a commonly used copyright protection method^48–51^. Copyright protection methods based on content retrieval and big data technology are mostly used to monitor and analyze suspected infringing content, which is beneficial to infringement early warning^52,53^.

However, the traditional digital copyright protection process requires verification, processing, encryption and other procedures by a trusted copyright center before entering the market transaction link. The transaction procedures are cumbersome, and the management of copyright transaction data adopts a centralized storage method^50–52^. With the increasing demand of copyright transaction, the traditional transaction mode is inefficient. In the process of copyright transaction, the circulation of ownership is not clear, and the effective management of transaction records is lacking. At the same time, copyright owners still need to obtain authorization through third-party copyright agencies and cannot effectively connect with copyright purchasers directly, which can easily lead to opaque income, untraceable transactions^54,57,58^, rights disputes and other phenomena.

Blockchain is a distributed ledger^55,59–61^ that integrates key technologies such as distributed storage, point-to-point transmission^62^, consensus mechanism^63^, cryptographic algorithms and smart contracts^64,65^. Its unique decentralization and non-tampering characteristics provide new ideas for copyright transactions. Based on this, researchers have applied blockchain to copyright transaction management, using smart contracts to implement decentralized transaction processes and improve the efficiency of copyright transactions^66^. However, these studies ignore the data redactionredacting requirements brought by the change of ownership after the copyright transaction^67^. In addition, the storage of copyright transaction data also faces the bottleneck problem of node storage. The Chameleon Hash (CH) algorithm enables blockchain editability^68,69^. It uses chameleon hash and trapdoor key to replace the traditional collision resistant hashing algorithm. The party with the trapdoor key can find the collision without changing the hash output and breaking the hash link. Thus, the data on the blockchain can be modified. However, directly combining chameleon hash with blockchain does not consider the decentralization issue. Researchers have proposed chameleon hashing based on threshold secret sharing^64–66^, which distributes trapdoor keys to multiple nodes in the blockchain through secret sharing. It improves decentralization of data redaction to a certain extent, but chameleon hash key generation relies on a trusted center. In^66^, they have improved the existing chameleon hash algorithm and improves the decentralization degree of the blockchain data redaction process. Among them, multiple nodes hold the sub-keys of chameleon hash and cooperate to recover the system private key. The scheme uses the asymmetric encryption of the blockchain to protect the confidentiality of the private key, but in the scenario of the gradual increase of permission nodes, the exit of the node requires large time overhead, and the lack of node incentive mechanism is not conducive to the flexible control of permission nodes. So, it’s effective in ensuring data confidentiality of the copyright transferor, resistance to malicious nodes, and the confidentiality and accountability of the private key of the chameleon hash in the IoT environment and Blockchain based IoT environment too.

In order to solve the problem that blockchain-based copyright transaction cannot perform ownership change and effectively connect with copyright registration, we have studied and improved the blockchain data redacting method based on distributed multi-center chameleon hash. Combined with the attribute-based cryptography, we have designed a permission node exit and change mechanism, which reduces the time overhead in the process of private key broadcast when the permission node quits, improves the flexibility of the permission node selection, and thus improves the enthusiasm of nodes to participate in data redactionredacting. At the same time, the waste of blockchain node storage resources is reduced.

Our contributions are as follows:

(1) A blockchain-based copyright transaction model and scheme supporting data redaction are proposed. The participants of the model include the copyright transferor, the copyright transaction purchaser, and the transaction verifier. When the copyright transaction is verified by the verifier, the copyright transferor and the purchaser complete the automatic copyright transaction process through the smart contract. At the same time, the copyright registration data redacting is supported to further save the storage space of copyright data.

(2) The blockchain data redacting method based on distributed multi-center chameleon hash is studied and optimized, and an improved permission node selection, exit and change mechanism is proposed to reduce the time overhead of chameleon hash sub-private key distribution in multi-node scenarios. It flexibly controls the selection of permission nodes, and improves the enthusiasm of nodes to participate in data redaction.

(3) The sub-private key security protection method based on KEA-CPABE-UK is proposed. The confidentiality of the sub-private key in the broadcast process is guaranteed by KEA-CPABE-UK algorithm, and the accountability mechanism is introduced to track the identity of the malicious node who leaks the key. The risk of chameleon hash private key caused by user key leakage is reduced by key update mechanism.

The rest of this paper is arranged as follows: Sect.  2 has described about the copyright transaction model based on blockchain. Section  3 has described the design of the copyright transaction scheme supporting data editable on blockchain in detail. In Sect.  4, the security performance analysis and experimental analysis of the proposed scheme are discussed in depth. Section  5 has concluded our work.

## Blockchain-based copyright transaction model

The copyright transaction model described in this section is relied on the blockchain network and smart contracts to achieve decentralized, fair, and autonomous transactions. It allows copyright transferors and purchasers to conduct transactions through the blockchain network, and implement information query and data redacting services.

**Fig. 1:**
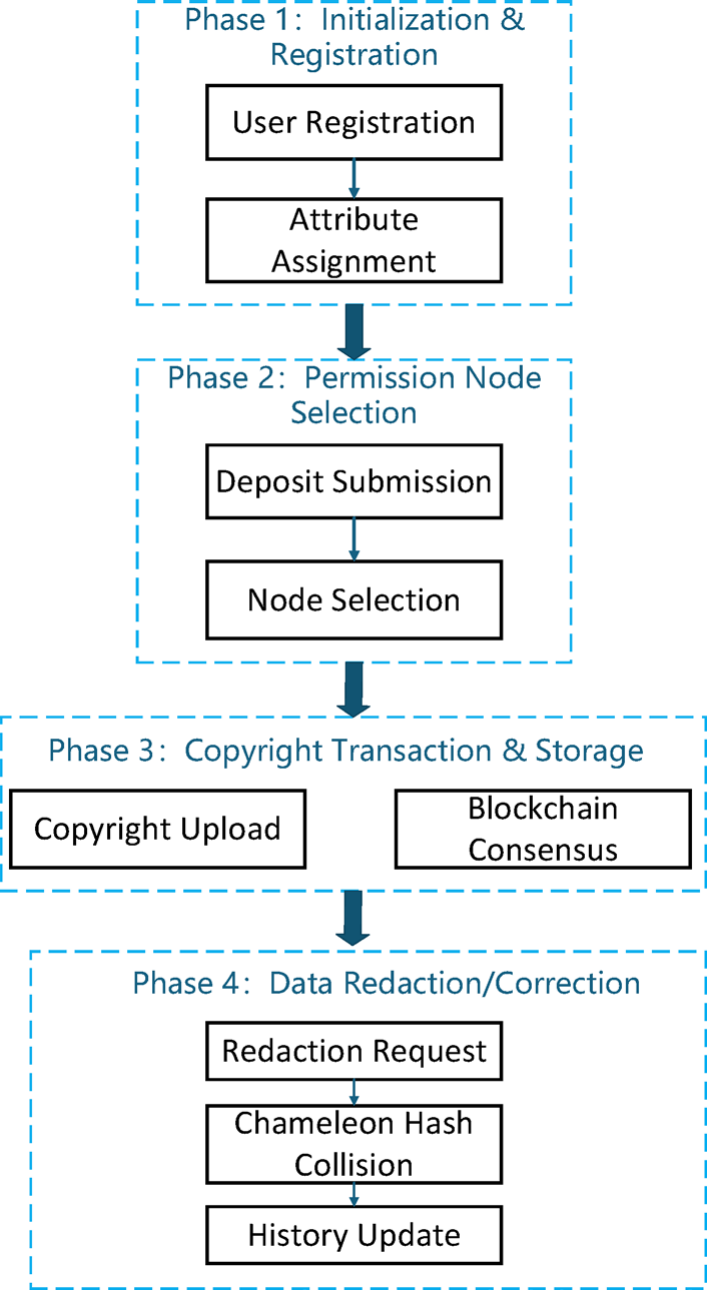
The graphical pipeline of the proposed multi-center copyright transaction and redaction framework.

As shown in Fig. 1, the system architecture is divided into four main phases: registration, node selection, transaction, and redaction. In the Registration Phase, users obtain identity credentials and attribute keys from the RA and AA. The Selection Phase involves the admission of permission nodes based on deposit staking and reputation evaluation. During the Transaction Phase, digital copyright information is encrypted and recorded on the blockchain through consensus. Finally, the Redaction Phase has utilized the multi-center chameleon hash mechanism to perform authorized data updates or error corrections while maintaining the structural integrity of the blockchain.

**Fig. 2:**
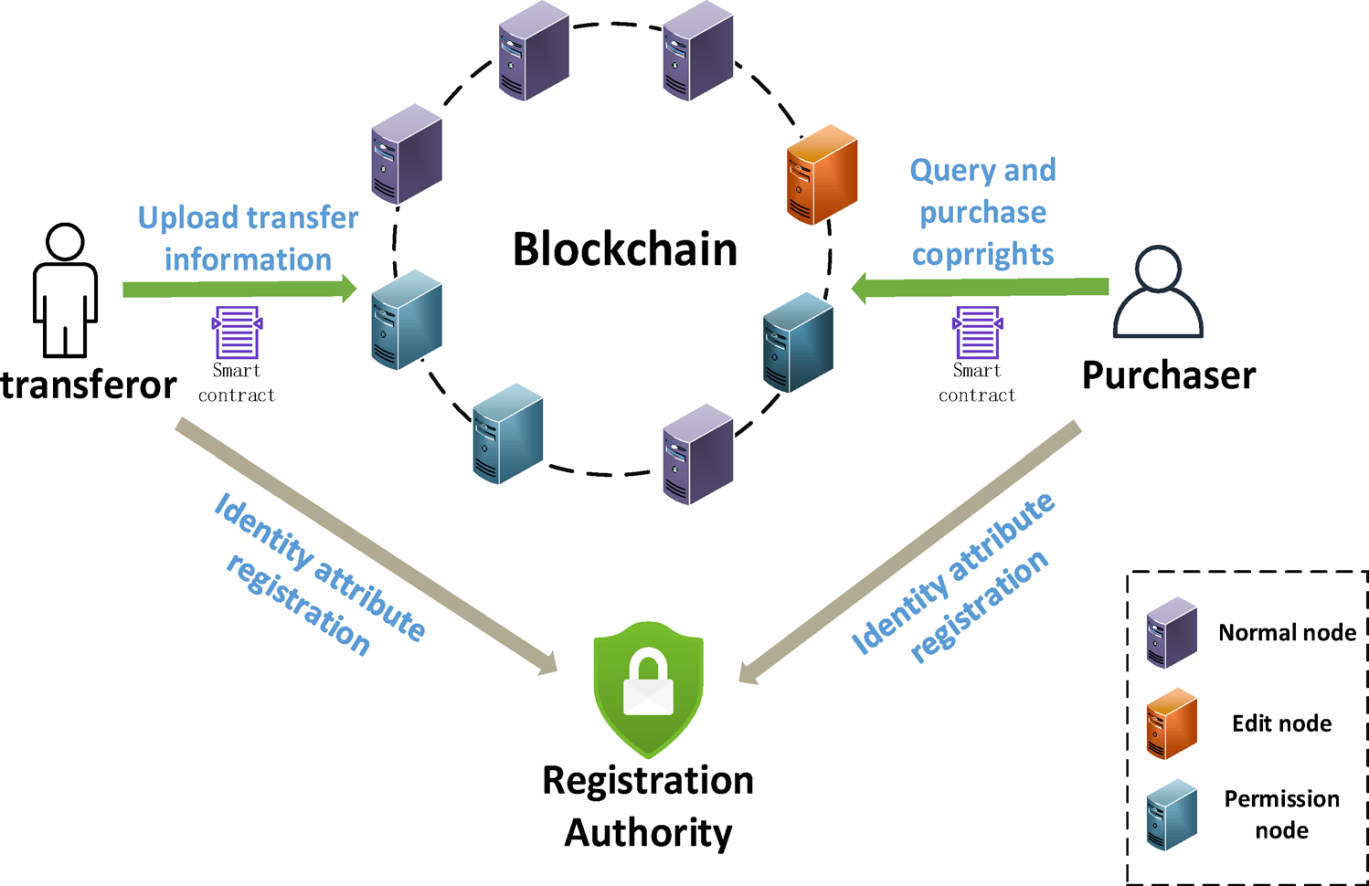
Copyright transaction model based on blockchain.

Figure 2 has shown the copyright transaction model based on blockchain. The participating entities and definitions of this model are as follows:

(1) Copyright transferor. The copyright transferor hopes to transfer the copyright through the blockchain copyright transaction model. It publishes the name of its work, copyright owner identity information, rights content, transfer price and other related terms to the blockchain network.

(2) Copyright purchaser. Copyright purchasers can query copyright sales information and purchase copyrights. Once the information released by the copyright transferor meets the user’s expectations, they can conduct copyright transactions through the blockchain network and smart contracts to complete decentralized automatic payment and data redacting.

(3) Blockchain. We adopt the consortium chain in our model. All copyright transferors and copyright purchasers must be registered before they can join the blockchain for transactions. There are permission nodes, redacting nodes and ordinary nodes in the blockchain network. The nodes cooperate to assist copyright transferors and purchasers to conduct decentralized secure transactions and complete the redacting of copyright data.

(4) Registration Authority. The registration authority registers the identities of users joining the blockchain and distributes keys. Only users who have successfully registered can conduct copyright transactions in the blockchain network.

## Blockchain-based copyright transaction scheme

Based on the model described in Sect.  2, this section has introduced the detailed design of a blockchain copyright transaction scheme that supports data editability. It includes several stages: initialization, permission nodes selection, copyright information upload, copyright transaction, copyright data redactionredacting, permission nodes exit, and permission nodes change.

### Blockchain node type definition

In the blockchain copyright transaction model and scheme that we have designed to support data editability, the division of labor of nodes is refined. There are three types of nodes in the blockchain network. The types and definitions of nodes are shown in Table 1.

**Table 1:** Node types and definitions.

Node type	Node definition
Ordinary node	Receive transactions and verify the transaction data structure and signature integrity in the block
Permission node	Verify the identity of both parties to copyright transactions, participate in key generation during the data redactionredacting phase and verification of redacting proposals
Redacting node	Voted by permission nodes to execute the copyright data redactionredacting process

### Threat model and consensus protocol

In our proposed framework, we have adopted the Practical Byzantine Fault Tolerance (PBFT) consensus protocol, which is well-suited for consortium blockchains.

#### Threat Model: 

We have assumed a partially synchronous network. The Permission Nodes are modeled as honest-but-curious in the general verification phase but can be Byzantine in worst-case scenarios. We have assumed that at most $\:f$ permission nodes are malicious, where the total number of permission nodes $\:3f\:+\:1$. The Redacting Node is temporarily trusted during the redaction execution but is subject to strict accountability via digital signatures. External adversaries are assumed to have full control over the network channels but cannot break standard cryptographic primitives (e.g., discrete logarithm problem).

Specifically, we have formally defined the trust assumptions for the system entities as follows: The Registration Authority (RA) and Attribute Authority (AA) are assumed to be semi-trusted (honest-but-curious); they will honestly execute the designated protocols but may attempt to glean information about user attributes. Permission nodes are assumed to be rational and potentially malicious. We have defined an adversary capable of controlling up to $\:f$ malicious permission nodes, where the total number of permission nodes is $\:3f\:+\:1$. The adversary’s capabilities include attempting strategic collusion among permission nodes, attempting to compromise attribute keys, and exhibiting malicious behavior during the node exit or data redaction phases. The Redacting Node is temporarily trusted during the redaction execution but is subject to strict accountability via digital signatures.

### Design of copyright transaction method based on Blockchain

This section has described the blockchain-based copyright transaction process. The specific steps are as follows:

(1) Initialization.

First, select the multiplicative group $\:{Z}_{p}^{\mathrm{*}}$, whose generator is $\:{g}_{1}$; $\:p\:$is a prime number, and $\:p=kq+1$. Enter the security parameter $\:\lambda\:$ and generate the chameleon hash public parameter $\:{pp}_{1}=\left\{g,p,q\right\}$. Then input the attribute set $\:U$ of the system, select the security parameter $\:{\lambda\:}^{{\prime\:}}$, and output the system public parameter $\:{pp}_{2}$ and the master key $\:MSK$.

(2) Attribute registration.

Before joining the blockchain network, a node first registers with the attribute registration agency and submits its own attribute set. The attribute registration agency generates the node’s private key $\:{SK}_{u}$ based on the node attribute $\:\left\{{A}_{i}\right\}$ and the system master key $\:MSK$, and sends it to the registered node.

(3) Permission node selection.

In the initial state, the system first sets the access structure $\:\left(M,\rho\:\right)$ according to the user attributes of the copyright blockchain, where $\:M$ is a matrix of size $\:m\times\:n$ and $\:\rho\:$ is a mapping function.

Select $\:K$ permission nodes that conform to the access structure. Each permission node needs to submit a deposit to the smart contract. Permission nodes have the right to choose to exit. When there are too many exit nodes or there are permission nodes doing evil, the system will change or re-select the permission nodes.

(4) Permission node key generation.

Each permission node generates its own chameleon hash sub-private key. For the permission node $\:{E}_{i}$, the sub-private key $\:{sec}_{i}$, $\:{(sec}_{i}\in\:{Z}_{q}^{\mathrm{*}})$ is randomly generated. Then use the public parameter $\:{pp}_{1}$ to generate the respective chameleon hash public key $\:{pub}_{i}={g}^{{sec}_{i}}modq$.

(5) Copyright transaction.

When a registered copyright owner wishes to transfer, he first needs to publish the copyright information to be transferred to the blockchain. The copyright transferor sends a transfer transaction request to the blockchain, and the permission node verifies the identity of the copyright transferor and the legality of the transaction. If the verification is successful, the transaction request is signed and broadcast to the blockchain network. Other nodes verify the integrity of transaction data structures and signatures.

The transaction information of the copyright transferor is:

1 $$\:\begin{array}{c}Transfer=\left\{ID,\:ADD,BlockNo,\:TransNo,Price,Other\right\}\end{array}$$

Among them, $\:ID$ is the work identification, $\:ADD$ is the address of the copyright transferor, $\:BlockNo$ is the block number where the copyright registration information is located, $\:TransNo$ represents the transaction number where the copyright registration information of the work is located, $\:Price$ represents the transfer price, and $\:Other$ is other terms. After successful verification, the permission node signs the $\:Transfer$, that is, $\:{Sig}_{transfer}$. When copyright transaction users inquire and need to purchase the corresponding copyright, they conduct transactions with the copyright transferor through the deployed smart contract to ensure that the copyright purchaser successfully obtains the copyright and the copyright transferor can obtain corresponding benefits.

(6) Data Redaction.

Copyright transactions need to ensure that the copyright purchaser successfully obtains the copyright. After the copyright purchaser makes payment according to the contract, the system starts the data redacting process. Among them, the copyright registration information has been packaged and uploaded to the chain using the chameleon hash function, so on-chain copyright data redacting based on the chameleon hash is supported.

Assume that the copyright registration data needs to be modified from $\:d$ to $\:{d}^{{\prime\:}}$ after the copyright transaction. The system selects an permission node $\:l$, which sends a copyright registration data redactionredacting proposal $\:DataRedaction$ to the blockchain network:

2 $$\:DataRedaction=\left\{{ID}_{l},TransferNo,BlockNo,\:TransactionNo,\:d,{d}^{{\prime\:}},{Sig}_{l}\right\}$$

Among them, $\:{ID}_{l}$ is the identity of the proposal initiator $\:l$, $\:TransferNo$ is the copyright transfer transaction number, $\:BlockNo$ is the block number where the data to be edited is located, $\:TransactionNo$ is the transaction number where the data to be edited is located, $\:d$ is the original data before redacting, $\:{d}^{{\prime\:}}$ is the edited data, and $\:{Sig}_{l}$ is the signature of the proposal by the permission node.

All other permission nodes verify the $$\:DataRedaction$$ proposal. To ensure liveness and scalability, we have adopted a threshold signature scheme. If more than 2/3 of the permission nodes (i.e., a quorum in PBFT) agree to the edit, the system selects the edit node $r$ based on the voting results. Each permission node $$\:{E}_{i}$$ encrypts and broadcasts its own chameleon hash private key $$\:{sec}_{i}$$ to the redacting node $$\:r$$, and the redacting node $$\:r$$ calculates the chameleon hash private key $$\:s={sec}_{1}+{sec}_{2}+{sec}_{3}+\cdots\:{+sec}_{k}$$. Then node $$\:r$$ executes the hash collision algorithm, obtains a new random number $$\:{r}^{{\prime\:}}$$, broadcasts the data redactionredacting proposal and the new random number $$\:{r}^{{\prime\:}}$$, and updates the local block after verification by the blockchain node. The edit node returns the edited copyright data $$\:RedactableCD$$ to the copyright purchaser:

3 $$\:RedactableCD=\left\{ID,\:{ADD}^{{\prime\:}},BlockNo,\:TransactionNo\right\}$$

Among them, $\:ID$ is the original work number, $\:{ADD}^{{\prime\:}}$ is the address of the user who has purchased the copyright, $\:BlockNo$ is the block number where the copyright information of the work is located, and $\:TransactionNo$ is the transaction number where the copyright information of the work is located. After the data redactionredacting is completed, the copyright transaction ends, and the blockchain stores the transaction information.

**Fig. 3:**
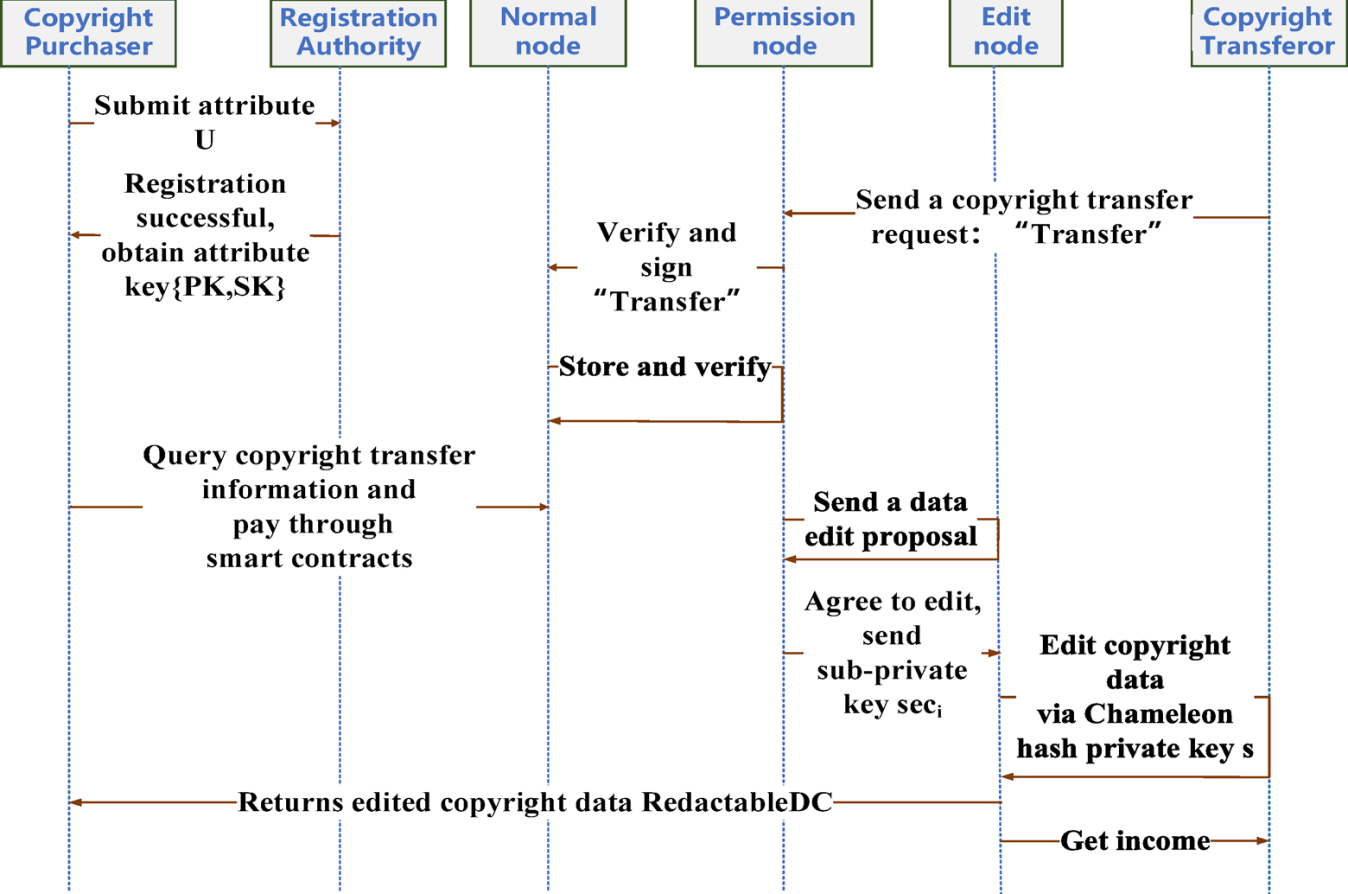
Main interaction process of blockchain copyright transaction method.

The main interaction process of the blockchain copyright transaction method that supports data editability is shown in Fig. 3:

### Editability of copyright data

In order to enable the copyright registration data to be modified, copyright data is stored on chain by chameleon hash. The block-level chameleon hash function can achieve the purpose of data redactionredacting, but this method will destroy the structure of the Merkle tree. The data of the previous and next blocks need to be modified. Therefore, this paper has adopted the transaction-based chameleon hashing method and improves the Merkle tree storage structure to support the editability of copyright data.

For example, $\:{T}_{i}=\left({O}_{i},{v}_{i}\right)$ is the $\:i-th$ transaction in block $\:{B}_{j}$. Among them, $\:{O}_{i}$ represents the address of the copyright registration user, and $\:{v}_{i}$ represents the work fingerprint. When copyright is traded, we need to modify the data in $\:{T}_{i}$, that is, change the original copyright owner’s address $\:{O}_{i}$ to the copyright purchaser’s address $\:{O}_{i}^{{\prime\:}}$. The sub-public key of the permission node is $\:{pub}_{i}$. In order to enable $\:{O}_{i}$ to be modified, $\:K$ permission nodes cooperate to generate the chameleon hash public key $\:Pub={pub}_{1}{pub}_{2}{pub}_{3}\dots\:{pub}_{K}$, and calculate the chameleon hash value for the leaf node data. Non-leaf nodes are constructed from the chameleon hash of leaf nodes.

**Fig. 4:**
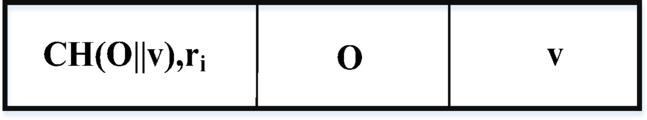
Editable leaf node structure.

Assume that there is a transaction $\:\left\{{T}_{1},{T}_{2},{T}_{3},{T}_{4},{T}_{5},{T}_{6},{T}_{7},{T}_{8}\right\}$ in a block $\:{B}_{j}$, and its corresponding user address $\:\left\{{O}_{i}\right\}$ is $\:\left\{\mathrm{3,5},\mathrm{6,7},\mathrm{9,13,15,16}\right\}$, then the leaf node structure of the Merkle tree is shown in Fig. 4.

**Fig. 5:**
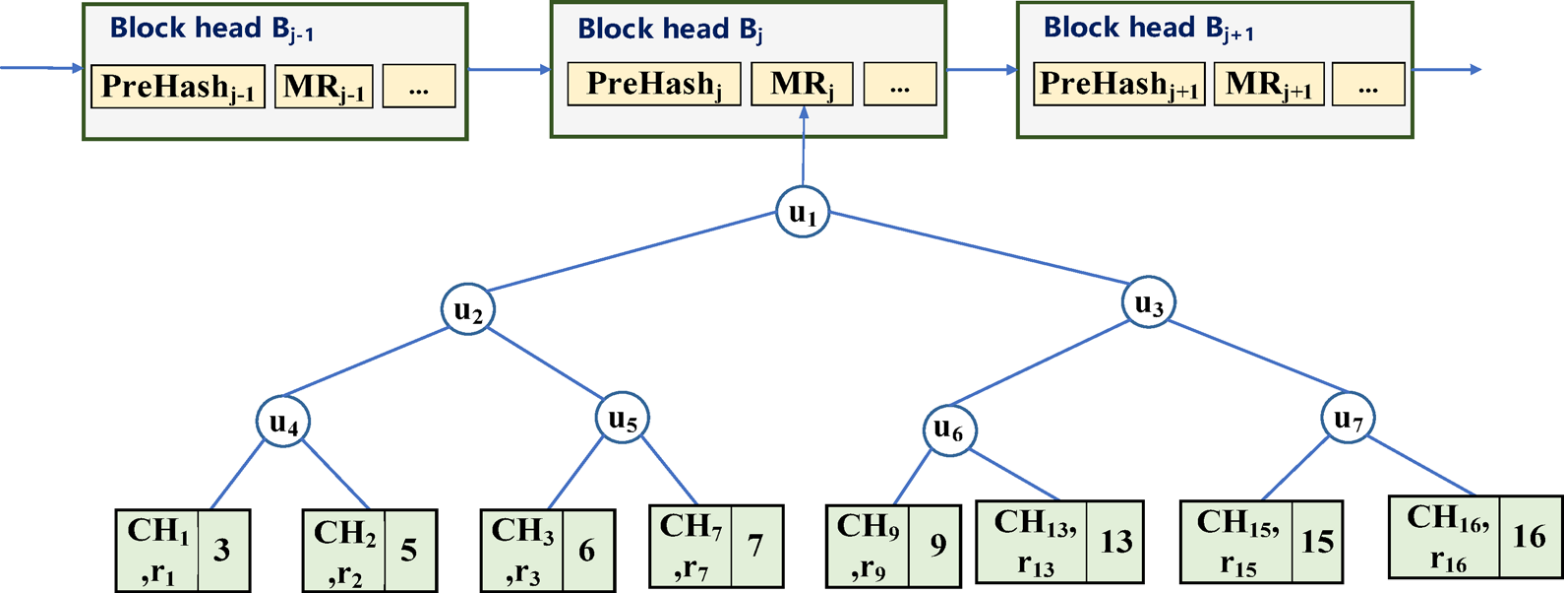
Blockchain storage structure supporting data redactionredacting.

**Fig. 6:**
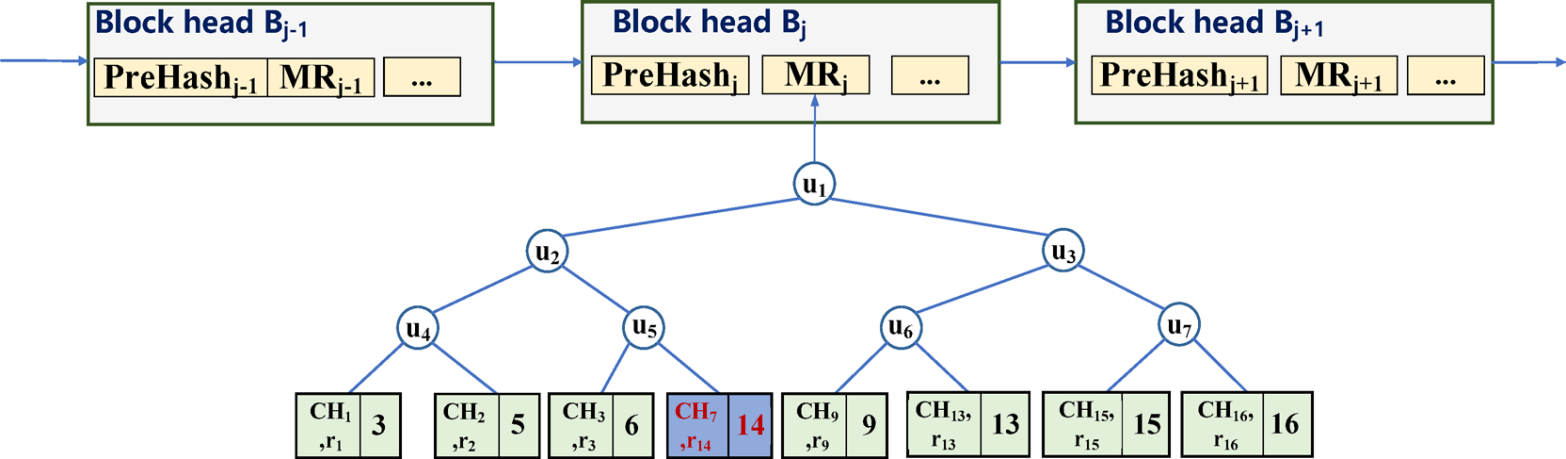
Blockchain storage structure after data redacting.

The blockchain storage structure that supports data redactionredacting is shown in Fig. 5. When data $\:{O}_{i}$ needs to be modified (for example, user address 7 is changed to 14), the redacting node first copies the data of the node where 7 is located, then deletes the node where 7 is located, and then inserts a new node with the user address of 14. The redacting node uses the generated chameleon hash private key $\:s$ to calculate a new random value $\:{r}_{14}$, and stores the node value $\:\left\{{CH}_{7},{r}_{14},14\right\}$ after the transaction, so that after the copyright transaction the block structure remains unchanged. At this point, the redacting node completes the modification task, and the edited block structure is shown in Fig. 6.

Proof of Merkle Root Consistency:

Let $\:{H}_{ch}(\cdot )$ be the chameleon hash function. For a leaf node representing transaction $\:{T}_{i}$, its value is $\:{L}_{i}={H}_{ch}\left({O}_{i},{v}_{i},r\right)$, where $\:r$ is the random parameter. When modifying data from $\:{O}_{i}$ to $\:{{O}^{{\prime\:}}}_{i}$, the redacting node uses the trapdoor key to find a collision $\:{r}^{{\prime\:}}$ such that: $\:{H}_{ch}\left({O}_{i},{v}_{i},r\right)={H}_{ch}\left({{O}^{{\prime\:}}}_{i},{v}_{i},{r}^{{\prime\:}}\right)$. Since the output hash value $\:{L}_{i}$ remains identical ($\:{L}_{i}$ = $\:{{L}^{{\prime\:}}}_{i}$), the hash values of all parent nodes in the Merkle tree up to the Merkle Root remain unchanged. Thus, the block integrity is preserved locally without requiring a hard fork or rewriting historical blocks.

### Selection, exit and change of permission nodes

Since permission nodes play an important role in blockchain-based copyright transactions, if there are malicious nodes among them, data redacting may be interrupted, and the copyright transaction may fail. Therefore, in order to prevent permission nodes from doing evil, we design a flexible and controllable permission node selection, exit and change mechanism.

(1) Permission node selection.

In the initial situation, the system can set the access structure$\:\left(M,\rho\:\right)$ based on the attributes $\:\left\{{A}_{i}\right\}$ of each node, the node computing capabilities, etc. $\:\left(M,\rho\:\right)$ is the access structure based on LSSS, where $\:M$ is the access matrix, and the function $\:\rho\:$ map each row of matrix $\:M$ to an attribute. Therefore, adding nodes that conform to the access structure to the system’s permission node set $\:\left\{{E}_{i}\right\}$ can make the selection of permission nodes based on specific conditions, ensuring the security and efficiency of the copyright transaction process to a certain extent. In order to further prevent permission nodes from doing evil, each permission node first needs to submit a deposit $\:{EM}_{i}$ as collateral. As shown in (4), the deposit $\:{EM}_{i}$ is related to the number of sub-private keys $\:{sec}_{i}$ held by the permission node, the length of time it serves as the permission node, and the number of times it completes the data redactionredacting task.

4 $$\:\begin{array}{c}{EM}_{i}=ntd\cdot w\end{array}$$

where $\:w$ is the amount of a single deposit, $\:n$ is the number of sub-private keys held by the permission node, $\:t$ is the length of time it has served as the permission node, and $\:d$ is the number of times it has completed data redactionredacting tasks.

(2) Permission node exit mechanism.

The exit of the permission node may affect the copyright transaction process and cause the transaction to fail. Therefore, we designed a method for the secure exit of permission nodes. Through the incentive mechanism and deposit mechanism, on the one hand, it increases the enthusiasm of nodes to join permission nodes, on the other hand, it increases the cost of nodes doing evil and prevents nodes from committing malicious acts. Assume that the permission node $\:{E}_{i}$ chooses to exit, the specific steps are:

① The permission node $\:{E}_{i}$ first sends an exit request message $\:ExitRequest$ to the system. After receiving it, other permission nodes confirm the message and start the permission node exit mechanism. The permission node $\:{E}_{i}$ encrypts the sub-private key $\:{sec}_{i}$ it controls through the public parameter $\:PP$ of the KEA-CPABE-UK algorithm and broadcasts:

5 $$\:\begin{array}{c}Encrypt\:\left(PP,\left(M,\rho\:\right){sec}_{i}\right)\to\:CT\end{array}$$

② After other permission nodes obtain the $\:CT$, they use their own attribute private key $\:SK$ to decrypt the $\:CT$ and obtain the sub-private key $\:{sec}_{i}$:

6 $$\:\begin{array}{c}Decrypt\:\left({SK}_{u},CT\right)\to\:{sec}_{i}\end{array}$$

③ After obtaining the sub-private key $\:{sec}_{i}$ of the permission node, verify the correctness of $\:{sec}_{i}$:

7 $$\:\begin{array}{c}Verify\left({sec}_{i},\:{pub}_{i}\right)\to\:1\:or\perp\:\end{array}$$

Verify whether $\:{pub}_{i}={g}^{{sec}_{i}}modq$ is established, where $\:{sec}_{i}\in\:{Z}_{q}^{\mathrm{*}}$, $\:{pub}_{i}$ represents the chameleon hash public key of $\:{E}_{i}$. If the verification algorithm passes, the system agrees that node $\:{E}_{i}$ exits and broadcasts the message, and the permission node $\:{E}_{i}$ gets back its mortgaged deposit $\:{EM}_{i}$.

The system gives $\:{E}_{i}$ certain incentives based on the time it has served as a permission node, the number of times it has completed data redactionredacting tasks, etc.

8 $$\:\begin{array}{c}{Reward}_{i}=td\cdot w\end{array}$$

Among them, $\:w$ is the amount of a single reward, and its value is the same as a single deposit; $\:t$ is the length of time it serves as a permission node, and $\:d$ is the number of times the node has completed data redactionredacting tasks.

④If the permission node does not hand over the sub-private key it holds or behaves dishonestly, the node’s reward $\:{Reward}_{i}$ will be zero and the deposit $\:{EM}_{i}$ will not be returned.

(3) Permission node change mechanism.

Since we use a multi-centralized Chameleon hash function in the data redactionredacting phase, the number of permission nodes is related to the degree of decentralization of chameleon hash key generation. The greater the number of nodes, the more decentralized key generation is. Therefore, when too many permission nodes exit, key generation will tend to be centralized. In extreme cases, all permission nodes may exit, making copyright transactions impossible.

Therefore, when the number of permission nodes is lower than a certain threshold, the system starts the permission node change mechanism. According to the current situation, a certain number of nodes that meet the requirements are selected from the blockchain nodes that apply to join the permission node. That is, according to the current access structure $\:\left(M,\rho\:\right)$, the node that meets the attribute requirements is selected to become the permission node. When there is no qualified permission node in the system, the access structure $\:{\left(M,\rho\:\right)}^{{\prime\:}}$ is reset based on the current node attributes, and $\:k$ permission nodes that meet the current access structure $\:{\left(M,\rho\:\right)}^{{\prime\:}}$ are selected.

In addition, there may be permission nodes in the system to do evil, such as:

The permission node broadcasts the wrong sub-private key, causing the edit node key generation to fail;

The permission node does not participate in the transaction process, causing the copyright transaction to fail;

The permission node maliciously leaks the attribute key, causing a security threat to the chameleon hash private key.

Once the above situation occurs, the system will revoke the permissions of the permission node, and will no longer assist the node in updating the key when the next time segment comes, and will ban the malicious node.

It is important to note that the proposed incentive and penalty mechanisms are relied on a heuristic economic model. While the required deposit $\:{EM}_{i}$ strictly increases the financial cost of malicious behavior and the reward incentivizes honest participation, we have not conducted a formal game-theoretic analysis to prove that this structure perfectly discourages all rational but selfish behavior (e.g., strategic collusion among nodes where the payout of an attack exceeds the lost deposit). We have identifdi this lack of a formal incentive analysis as a limitation of the current work. Future research will focus on a semi-formal incentive analysis to optimize parameter sensitivity (e.g., the precise ratio of deposit size to potential attack gain) in zero-trust environments.

### Sub-private key security protection based on KEA-CPABE-UK

In supporting editable blockchain copyright transactions, the sub-private key of the chameleon hash is critical to the blockchain’s data redacting. Malicious nodes or attribute agencies may leak keys, posing a threat to the security of chameleon hash private keys. Due to the ambiguity of attribute encryption, the identity of the key leaker is difficult to trace. This paper proposes a Key Exposure Accountable Ciphertext Policy Attribute based Encryption with Updatable Keys (KEA-CPABE-UK) algorithm that supports key updates to ensure confidentiality during the broadcast process of sub-private keys. Among them, the node key update mechanism and accountability mechanism are introduced to track the identity of the malicious node or organization that leaked the key, reduce the risk of the sub-private key after the node attribute key is leaked, and strengthen the security protection of the sub-private key. At the same time, the possibility of nodes doing evil is further reduced.

(1) KEA-CPABE-UK algorithm construction.

#### $\:Setup\left(\lambda\:\right)\to\:\left(PP,MSK\right)$

Given the security parameter $\:\lambda\:$, output the public parameter $\:PP$ and the system master key $\:MSK$. The specific work is as follows.

①The attribute mechanism $\:AA$ selects two multiplicative cyclic groups $\:{G}_{1}$, $\:{G}_{2}$, and $\:g$ with prime order $\:p$ as the generators of $\:{G}_{1}$. Define a bilinear mapping $\:e:{G}_{1}\times\:{G}_{1}\to\:{G}_{2}$.

②Select $\:\alpha\:,l,x\in\:{Z}_{P}$, and select $\:{u}_{i}\in\:{Z}_{p}^{\mathrm{*}}$ for each attribute in the node attribute set $\:\left\{{A}_{i}\right\}$. Calculate $\:Y={e\left(g,g\right)}^{\alpha\:}$, $\:L={g}^{l}$, $\:X={g}^{x}$, $\:{U}_{i}={g}^{{u}_{i}}$. Define the hash function $\:{H}_{1}:{\left\{\mathrm{0,1}\right\}}^{\mathrm{*}}\to\:{G}_{1}, {H}_{2}:{\left\{\mathrm{0,1}\right\}}^{\mathrm{*}}\to\:{Z}_{p}^{\mathrm{*}}$.

③Based on the above calculation, $\:AA$ outputs public parameters $\:PP=\left\{{G}_{1},{G}_{2},e,p,g,Y,L,X,{U}_{i},{H}_{1},{H}_{2}\right\}$, and master key $\:MSK=\left\{{u}_{i},l,\alpha\:,x\right\}$.

#### $\:SigGen\left(PP\right)\to\:\left({SK}_{SIG},{PK}_{SIG}\right)$

Input the public parameter $\:PP$ and output the key pair $\:\left({PK}_{SIG},{SK}_{SIG}\right)$. The specific work is as follows.

The node randomly selects $\:{q}_{u}\in\:{Z}_{p}^{\mathrm{*}}$ and generates a signature key pair $\:\left({PK}_{SIG},{SK}_{SIG}\right)$, where $\:{SK}_{SIG}={q}_{u}$, $\:{PK}_{SIG}={g}^{{q}_{u}}$. The node saves $\:{SK}_{SIG}$ and makes $\:{PK}_{SIG}$ public.

#### $\:KeyGen\left(PP,MSK,\left\{{A}_{i}\right\},UID\right)\to\:{SK}_{u}$

Input public parameter $\:PP$, master key $\:MSK$, node attribute set $\:\left\{{A}_{i}\right\}$, node identity identifier $\:UID\in\:{Z}_{p}^{\mathrm{*}}$, and output node private key $\:{SK}_{u}$. The specific work is as follows.

① Node $\:u$ applies for a private key from $\:AA$ and submits its own attribute set $\:\left\{{A}_{i}\right\}$ and identity identifier $\:UID$.

② Let $\:l$ be the facilitator key.

③ $\:AA$ randomly selects $\:t\in\:{Z}_{p}^{\mathrm{*}}$, calculates $\:{K}_{0}={g}^{t}$, and sends $\:{K}_{0}$ to node $\:u$ through the secure channel.

④ $\:u$ signs after receiving $\:{K}_{0}$, calculates $\:{\sigma\:}_{u}={{H}_{1}\left({K}_{0},\:{PK}_{SIG}\right)}^{{SK}_{SIG}}$, and sends $\:{\sigma\:}_{u}$ secretly to $\:AA$. $\:AA$ verifies the validity of $\:{\sigma\:}_{u}$, that is, whether $\:e\left({\sigma\:}_{u},g\right)=e\left({H}_{1}\left({K}_{0},\:{PK}_{SIG}\right),{PK}_{SIG}\right)$ is established.

⑤ $\:AA$ calculates $\:{K}_{1}={g}^{\alpha\:d+xt}$, $\:{K}_{i,0}={U}_{i}^{t}{{H}_{1}\left({A}_{i},{T}_{0}\right)}^{l}$. Among them, $\:{T}_{0}$ is the initial time segment parameter, $\:d={H}_{2}\left({\sigma\:}_{u},{K}_{0},UID\right)$. Output the node’s initial private key $\:{SK}_{u,0}=\left\{{K}_{0},{K}_{1},{K}_{i,0},{\sigma\:}_{u},UID\right\}$, and save the node $\:UID$ and its initial private key information.

#### $\:KeyUpdate\left({A}_{i},{T}_{n-1},{T}_{n},l\right)\to\:{UP}_{i,{T}_{n}}$

Input attribute $\:{A}_{i}$, adjacent time segment parameters $\:{T}_{n-1}$, $\:{T}_{n}$, facilitator key $\:l$, output node key update components $\:{UP}_{i,{T}_{n}}$. The specific work is as follows.

The key assistant inputs $\:{A}_{i}, {T}_{n-1}, {T}_{n}, l$, and calculates the key update component $\:{UP}_{i,{T}_{n}}={\left(\frac{{H}_{1}\left({A}_{i},{T}_{n}\right)}{{H}_{1}\left({A}_{i},{T}_{n-1}\right)}\right)}^{l}$.

#### $\:NodeUpdate\left({UP}_{i,{T}_{n}},{SK}_{u,n-1}\right)\to\:{SK}_{u,n}$

Input the temporary private key $\:{SK}_{u,n-1}$ and the node key update component $\:{UP}_{i,{T}_{n}}$, and output the node’s new private key $\:{SK}_{u,n}$. The specific work is as follows.

Node input $\:{UP}_{i,{T}_{n}}, {SK}_{u,n-1}$, calculate $\:{K}_{i,n}={K}_{i,n-1}\cdot {UP}_{i,{T}_{n}}$, output the new private key $\:{SK}_{u,n}=\left\{{K}_{0},{K}_{1},{K}_{i,n},{\sigma\:}_{u},UID\right\}$ at time segment $\:{T}_{n}$.

#### $\:Encrypt\left({sec}_{i},\left(M,\rho\:\right),PP\right)\to\:CT$

Input the chameleon hash sub-private key $\:{sec}_{i}$, the access structure $\:\left(M,\rho\:\right)$ and the public parameter $\:PP$, and output the sub-private key ciphertext $\:CT$. The specific work is as follows.

①The exit node selects the secret value $\:s$ and the LSSS-based access structure $\:\left(M,\rho\:\right)$ for its sub-private key plaintext $\:{sec}_{i}$, where $\:M$ is an $\:m\times\:n$ matrix, and the function $\:\rho\:$ maps the attributes to the rows of the matrix $\:M$. Calculate $\:{C}_{0}={sec}_{i}{Y}^{s}$, $\:{C}_{1}={g}^{s}$.

②Select a random vector $\:\overrightarrow{v}=\left(s,{y}_{2},\dots\:,{y}_{n}\right)\in\:{Z}_{p}^{n}$. For $\:i=1\:to\:m$, let $\:{\lambda\:}_{i}=\overrightarrow{v}\cdot \:{M}_{i}$. Let $\:I\subset\:\left\{\mathrm{1,2},\dots\:,m\right\}$ be defined as $\:I=\left\{i:\rho\:\left(i\right)\in\:\left\{{A}_{i}\right\}\right\}$.

③ Randomly select $\:{r}_{1},{r}_{2},\dots\:,{r}_{n}\in\:{Z}_{p}^{\mathrm{*}}$, calculate $\:{C}_{1,i}={X}^{{\lambda\:}_{i}}{U}_{\rho\:\left(i\right)}^{-{r}_{i}}$, $\:{C}_{2,i}={g}^{{r}_{i}}$, $\:{C}_{3,i}={L}^{-{r}_{i}}$.

④ Based on the above calculation, the node outputs the ciphertext $\:CT=\left\{{C}_{0},{C}_{1},{C}_{1,i},\:{C}_{2,i},{C}_{3,i}\right\}$.

#### $\:Decrypt\left(CT,PP,{SK}_{u,n}\right)\to\:{sec}_{i}$

Input ciphertext $\:CT$, public parameter $\:PP$, node private key $\:{SK}_{u,n}$, and output sub-private key plaintext $\:{sec}_{i}$. The specific work is as follows.

After the permission node $\:u$ receives the sub-private key ciphertext $\:CT$ of the exit node, it performs the following decryption calculation, where $\:d={H}_{2}\left({\sigma\:}_{u},{K}_{0},UID\right), h={H}_{1}\left({A}_{i},{T}_{n}\right)$.

9 $$\:\begin{array}{c}{sec}_{i}={C}_{0}{\left(\frac{\prod\:_{i\in\:I}{\left(e\left({C}_{1,i},{K}_{0}\right)\cdot \:e\left({C}_{2,i},{K}_{\rho\:\left(i\right),n}\right)\cdot \:e\left({C}_{3,i},h\right)\right)}^{{\omega\:}_{i}}}{e\left({C}_{1},{K}_{1}\right)}\right)}^{\raisebox{1ex}{$1$}\!\left/\:\!\raisebox{-1ex}{$d$}\right.}\end{array}$$

(2) KEA-CPABE-UK security model.

The security model for the sub-private key confidentiality proof in the KEA-CPABE-UK algorithm is as follows:

Initialization: The adversary $\:{\mathcal{A}}_{v}$ declares a challenge access structure $\:{\gamma\:}^{\mathrm{*}}$.

Setup: Challenger $\:{C}_{H}$ executes the $\:Setup$ algorithm, sends the public parameters to $\:{\mathcal{A}}_{v}$, and saves the master key $\:MSK$.

Phase 1: In Phase 1, $\:{\mathcal{A}}_{v}$ can perform polynomial key query and key update query for the attribute set, and the attribute set $\:{A}_{i}$ it queries does not satisfy the challenge access structure $\:{\gamma\:}^{\mathrm{*}}$.

Challenge: $\:{\mathcal{A}}_{v}$ sends two equal-length plaintext messages $\:{M}_{0}$ and $\:{M}_{1}$ to $\:{C}_{H}$. Subsequently, $\:{C}_{H}$ tosses a coin $\:\beta\:\in\:\left\{\mathrm{0,1}\right\}$, encrypts the message $\:{M}_{\beta\:}$ by challenging the access structure $\:{\gamma\:}^{\mathrm{*}}$, and sends the ciphertext to $\:{\mathcal{A}}_{v}$.

Phase 2: The adversary $\:{\mathcal{A}}_{v}$ repeats the query operation of Phase 1, and the attribute set it queries does not satisfy the challenge access structure $\:{\gamma\:}^{\mathrm{*}}$.

Guess: The adversary $\:{\mathcal{A}}_{v}$ outputs a guess $\:{\beta\:}^{\mathrm{*}}$ for $\:\beta\:$. $\:{\mathcal{A}}_{v}$ wins the game only when $\:{\beta\:}^{\mathrm{*}}=\beta\:$. Define $\:{\mathcal{A}}_{v}$’s advantage in this attack game as:

10 $$\:\begin{array}{c}Adv\left({\mathcal{A}}_{v}\right)=\left|{Pr}\left({\beta\:}^{*}=\beta\:\right)-\raisebox{1ex}{$1$}\!\left/\:\!\raisebox{-1ex}{$2$}\right.\right|\end{array}$$

Compared to existing accountable attribute-based encryption (A-ABE) schemes that primarily focus on white-box tracing, our KEA-CPABE-UK is tailored for redacting scenarios. It links ephemeral trapdoor reconstruction to the node’s identity with lower overhead, providing a practical balance between traceability and performance in IoT environments.

## Experiment and analysis

### Security performance

According to the design goals of our scheme, the security of blockchain copyright transactions is mainly reflected in the confidentiality of the copyright transferor’s private information, resistance to node maliciousness, confidentiality and accountability of the chameleon hash private key. The specific analysis is shown in Table 2.

**Table 2:** Security analysis of our scheme.

Security features	Analysis
Confidentiality of the copyright transferor’s privacy information	The private information of the transferor in the transfer information needs to be encrypted. Copyright transaction users will not obtain any private information of the copyright transferor without payment.
Malicious node detection	Resistance to malicious node behavior means that the chameleon hash algorithm ensures that permission nodes cannot edit blockchain data without obtaining all chameleon hash sub-private keys; in addition, the permission node selection and exit method uses a deposit-based reward and punishment mechanism and KEA-CPABE-UK algorithm to reduce the possibility of nodes doing evil.
Confidentiality of chameleon hash sub-private keys	During the exit phase of the permission node, the sub-private key is encrypted through the attribute key when broadcast. The security of the KEA-CPABE-UK algorithm ensures that non-authorized nodes cannot obtain the chameleon hash sub-private key, preventing the blockchain data from being illegally modified.
Accountability	After the transaction is successful, the permission node will sign the edit proposal. Its non-repudiation ensures the accountability of the initiator of the redacting proposal. In addition, the KEA-CPABE-UK algorithm used in the chameleon hash sub-private key broadcast phase provides accountability to malicious nodes and $\:AA$.

**Discussion on Key Security**:

Regarding the redacting node’s possession of the full ephemeral private key $\:s$: The key $\:s$ is reconstructed only for the specific transaction round and is tied to the specific DataRedaction proposal.

The non-repudiation property of the digital signature $\:{Sig}_{l}$ ensures that if the redacting node performs unauthorized edits, it can be mathematically traced and penalized (slashed) by the consortium. Furthermore, regarding the KEA-CPABE-UK model, while Chosen-Ciphertext Attack (CCA) security provides stronger guarantees, Chosen-Plaintext Attack (CPA) security is generally considered sufficient for this broadcast scenario where the primary threat is unauthorized access to the key rather than malleability of the ciphertext. Future work will explore upgrading to CCA-secure schemes for higher threat environments.

This solution needs to ensure the confidentiality of the chameleon hash sub-private key. Once the sub-private key is leaked, it will have a serious impact on blockchain copyright transactions. This section first verifies the correctness and accountability of the KEA-CPABE-UK algorithm, and uses the secure reduction method to securely prove the confidentiality of the node private keys in the KEA-CPABE-UK algorithm.

(1) Proof of correctness.

If $\:{\{\lambda\:}_{i}\}$ is a valid share of any secret value $\:s$, then there exists $\:\left\{{\omega\:}_{i},i\in\:I\right\}$ such that $\:\sum\:_{i\in\:I}{\omega\:}_{i}{\lambda\:}_{i}=s$. The decryption calculation process is as follows:

11 $$\begin{aligned} &\frac{\prod\:_{i\in\:I}{\left(e\left({C}_{1,i},{K}_{0}\right)\cdot e\left({C}_{2,i},{K}_{{\rho\:}_{\left(i\right),n}}\right)\cdot e\left({C}_{3,i},h\right)\right)}^{{\omega\:}_{i}}}{e\left({C}_{1},{K}_{1}\right)}\nonumber\\&=\frac{\prod\:_{i\in\:I}{\left(e\left({X}^{{\lambda\:}_{i}}{U}_{\rho\:\left(i\right)}^{-{r}_{i}},{g}^{t}\right)\cdot \:e\left({g}^{{r}_{i}},{U}_{\rho\:\left(i\right)}^{t}{{H}_{1}\left({A}_{i},{T}_{n}\right)}^{l}\right)\cdot \:e\left({L}^{-{r}_{i}},{H}_{1}\left({A}_{i},{T}_{n}\right)\right)\right)}^{{\omega\:}_{i}}}{e\left({g}^{s},{g}^{\alpha\:d+xt}\right)}\nonumber\\ &=\frac{\prod\:_{i\in\:I}{\left(e\left({g}^{x{\lambda\:}_{i}},{g}^{t}\right) \cdot e\left({g}^{-{r}_{i}{u}_{\rho\:\left(i\right)}},{g}^{t}\right)\cdot \:e\left({g}^{{r}_{i}},{g}^{{u}_{\rho\:\left(i\right)}t}\right)\cdot \:e\left({g}^{{r}_{i}},{{H}_{1}\left({A}_{i},{T}_{n}\right)}^{l}\right)e\left({L}^{-{r}_{i}},{H}_{1}\left({A}_{i},{T}_{n}\right)\right)\right)}^{{\omega\:}_{i}}}{e\left({g}^{s},{g}^{\alpha\:d+xt}\right)}\nonumber\\&=\frac{\prod\:_{i\in\:I}{\left(e\left({g}^{x{\lambda\:}_{i}},{g}^{t}\right)\cdot e\left({g}^{{r}_{i}},{{H}_{1}\left({A}_{i},{T}_{n}\right)}^{l}\right)\cdot \:e\left({g}^{-{r}_{i}l},{H}_{1}\left({A}_{i},{T}_{n}\right)\right)\right)}^{{\omega\:}_{i}}}{e\left({g}^{s},{g}^{\alpha\:d+xt}\right)}\nonumber\\&=\frac{\prod\:_{i\in\:I}{\left(e\left({g}^{x{\lambda\:}_{i}},{g}^{t}\right)\right)}^{{\omega\:}_{i}}}{e\left({g}^{s},{g}^{\alpha\:d+xt}\right)}=\frac{{e\left({g}^{x},{g}^{t}\right)}^{\sum\:_{i\in I}{\lambda\:}_{i}{\omega\:}_{i}}}{e\left({g}^{s},{g}^{\alpha\:d+xt}\right)}\nonumber\\&=\frac{{e\left({g}^{x},{g}^{t}\right)}^{s}}{e\left({g}^{s},{g}^{\alpha\:d}\left)e\right({g}^{s},{g}^{xt}\right)}\nonumber\\ &=\frac{1}{e\left({g}^{s},{g}^{\alpha\:d}\right)}\end{aligned}$$

Let $$\:v=\frac{1}{e\left({g}^{s},{g}^{\alpha\:d}\right)}$$, then:

12 $$\:\begin{aligned}{C}_{0}{v}^{\raisebox{1ex}{$1$}\!\left/\:\!\raisebox{-1ex}{$d$}\right.}&={sec}_{i}{Y}^{s}\frac{1}{e\left({g}^{s},{g}^{\alpha\:}\right)}\nonumber\\&={sec}_{i}{e\left(g,g\right)}^{\alpha\:s}\frac{1}{e\left({g}^{s},\:{\:g}^{\alpha\:}\right)}\nonumber\\&={sec}_{i}\end{aligned}$$

(2) Accountability for key leaks.

When a malicious node or $\:AA$ leaks the node’s private key, this method provides an accountability mechanism to track the identity of the key leaker. Let $\:{SK}_{u,{T}_{n}}^{{\prime\:}}=\left\{{K}_{0}^{{\prime\:}},{K}_{1}^{{\prime\:}},{K}_{i,n}^{{\prime\:}},{\sigma\:}_{u}^{{\prime\:}},{UID}^{{\prime\:}}\right\}$ be the exposed private key. The accountability mechanism works as follows:

If the suspicious node $\:{UID}^{{\prime\:}}$ is considered to be the key leaker, the accountability agency first verifies whether $\:e\left({K}_{1}^{{\prime\:}},g\right)={e\left(g,g\right)}^{\alpha\:d}\cdot \:e\left({g}^{a},\:{K}_{0}^{{\prime\:}}\right)$ is established;

②If the above equation is established, the accountability agency verifies whether $\:e\left({K}_{i,n}^{{\prime\:}},g\right)=e\left({K}_{0}^{{\prime\:}},{U}_{i}{h}^{l}\right)$ is established;

③If the above equation is established, the accountability agency verifies whether $\:e\left({\sigma\:}_{u}^{{\prime\:}},g\right)=e\left({H}_{1}\left({K}_{0}^{{\prime\:}},{PK}_{SIG}\right),{PK}_{SIG}\right)$ is established;

④If the above equations pass, the accountability agency outputs the node identity identifier $\:{UID}^{{\prime\:}}$ and determines that the node is the key leaker;

⑤If the node $\:{UID}^{{\prime\:}}$ provides the private key $\:{SK}_{u,{T}_{n}}^{{\prime\:}{\prime\:}}$ that can pass the above verification, denying that it is the leaker, the accountability agency determines that $\:AA$ is the key leaker.

(3) Security proof.

In this section, we use the Decisional Bilinear Diffie-Hellman (DBDH) problem to prove the confidentiality of the node’s private key in the KEA-CPABE-UK algorithm.

#### Theorem 6.1

If there is no polynomial-time adversary in the security model of the sub-private key confidentiality proof that can win the game with a non-negligible advantage$\:\:\varepsilon\:$, then in the KEA-CPABE-UK designed in this paper, the sub-private keys of nodes in the algorithm are confidential.

#### Theorem 6.2

If the DBDH problem on the $\:{G}_{2}$ group is intractable, then the node sub-private keys in the KEA-CPABE-UK algorithm are confidential.

#### Proof

Assuming that there is an adversary $$\:{\mathcal{A}}_{v}$$ that can break the confidentiality of the node private key in the KEA-CPABE-UK algorithm with a non-negligible advantage $$\:\epsilon\:$$ under the choice set model, then we can build a simulator $$\:\mathcal{B}$$ to solve the $$\:{G}_{2}$$ group with an advantage of $$\:\raisebox{1ex}{$\varepsilon\:$}\!\left/\:\!\raisebox{-1ex}{$2$}\right.$$ DBDH puzzle on. In the attack game, let $$\:{\mathcal{A}}_{v}$$ represent the adversary and $$\:{C}_{H}$$ represent the challenger. The simulator is constructed as follows:

Initialization: $\:{\mathcal{A}}_{v}$ declares a challenge access structure $\:{\gamma\:}^{\mathrm{*}}$.

Setup: $\:{C}_{H}$ executes the $\:Setup$ algorithm and selects two multiplicative cyclic groups $\:{G}_{1}$, $\:{G}_{2}$, and $\:g$ with prime order $\:p$ as the generators of $\:{G}_{1}$. Define a bilinear mapping $\:e:{G}_{1}\times\:{G}_{1}\to\:{G}_{2}$ and the in-game attribute set $\:{A}_{i}$. $\:{C}_{H}$ tosses a coin $\:\mu\:\in\:\left\{\mathrm{0,1}\right\}$, selects $\:a,b,c,z\in\:{Z}_{p}^{\mathrm{*}}$, and stipulates:

13 $$\:\begin{array}{c}\left\{\begin{array}{c}\left(A,B,C,Z\right)={({g}^{a},{g}^{b},{g}^{c},\:e\left(g,g\right)}^{abc}),\:\mu\:=0\:\\\:\left(A,B,C,Z\right)=\left({g}^{a},{g}^{b},{g}^{c},{e\left(g,g\right)}^{z}\right),\mu\:=1\end{array}\right.\end{array}$$

$\:{C}_{H}$ randomly selects $\:\left\{{u}_{i},l,\alpha\:,a\right\}\in\:{Z}_{p}^{\mathrm{*}}$, calculates $\:Y={e\left(g,g\right)}^{ab}$, $\:L={g}^{l}, X={g}^{x}, {U}_{i}={g}^{{u}_{i}}$, which implicitly sets $\:\alpha\:=ab$. Select $\:{H}_{1}:{\left\{\mathrm{0,1}\right\}}^{\mathrm{*}}\to\:{G}_{1}, {H}_{2}:{\left\{\mathrm{0,1}\right\}}^{\mathrm{*}}\to\:{Z}_{p}^{\mathrm{*}}$. The public parameters of the output system are $\:PP=\left\{{G}_{1},{G}_{2},e,p,g,Y,X,{U}_{i},L,{H}_{1},{H}_{2}\right\}$, and the master key $\:MSK=\left\{{u}_{i},l,\alpha\:,x\right\}$. $\:{C}_{H}$ sends $\:PP$ to $\:{\mathcal{A}}_{v}$, saving $\:MSK$.

Phase 1: $\:{\mathcal{A}}_{v}$ performs polynomial key query and key update query for the attribute set $\:{A}_{i}$, where $\:{A}_{i}$ does not satisfy the challenge access structure $\:{\gamma\:}^{\mathrm{*}}$. The inquiry steps are as follows:

Key generation query: $\:{\mathcal{A}}_{v}$ submits a key generation query about the attribute set $\:S$ to $\:\mathcal{B}$, and $\:\mathcal{B}$ responds as follows: $\:\mathcal{B}$ first queries the $\:{H}_{1}$ and $\:{H}_{2}$ oracles, and when calling the $\:{H}_{1}$ and $\:{H}_{2}$ functions, $\:\mathcal{B}$ will select the new elements $\:{i}^{\mathrm{*}}$, $\:{j}^{\mathrm{*}}$ from $\:{Z}_{p}^{\mathrm{*}}$, take $\:{g}^{{i}^{\mathrm{*}}}$, $\:{j}^{\mathrm{*}}$ as the output of the function respectively. $\:\mathcal{B}$ selects $\:t\in\:{Z}_{p}^{\mathrm{*}}$, calculates $\:{K}_{0}={g}^{t}$, $\:{K}_{1}={g}^{\alpha\:{d}^{\mathrm{*}}+xt}$. For each attribute $\:{A}_{i}$ in the attribute set $\:S$, $\:\mathcal{B}$ calculates $\:{K}_{i,0}={U}_{i}^{t}{g}^{l\left({{A}_{i}}^{\mathrm{*}}\cdot \:{{T}_{0}}^{\mathrm{*}}\right)}$, then the initial private key of $\:{\mathcal{A}}_{v}$ is expressed as $\:{SK}_{{u}^{\mathrm{*}},0}=\left\{{K}_{0},{K}_{1},{K}_{i,0},i\in\:S\right\}$. $\:{SK}_{{u}^{\mathrm{*}},0}$ is a valid initial private key because: $\:{K}_{0}={g}^{t}$, $\:{K}_{1}={g}^{\alpha\:{d}^{\mathrm{*}}+xt}$, $\:{K}_{i,0}={{U}_{i}^{t}{{H}_{1}\left({A}_{i},{T}_{0}\right)}^{l}=U}_{i}^{t}{g}^{l\left({{A}_{i}}^{\mathrm{*}}\cdot\:{{T}_{0}}^{\mathrm{*}}\right)}$.

Key update query: $\:{\mathcal{A}}_{v}$ sends a key update request for attribute set $\:S$ to $\:\mathcal{B}$, and its time segment parameters are $\:{T}_{n-1}$, $\:{T}_{n}$. $\:\mathcal{B}$ calculates $\:{UP}_{i,{T}_{n}}={g}^{l{{A}_{i}}^{\mathrm{*}}\left({{T}_{n}}^{\mathrm{*}}-{{T}_{n-1}}^{\mathrm{*}}\right)}$ and sends it to $\:{\mathcal{A}}_{v}$. The $\:{UP}_{i,{T}_{n}}$ produced by the above steps is a valid key update component, because:

14 $$\:\begin{array}{c}{UP}_{i,{T}_{n}}={\left(\frac{{H}_{1}\left({A}_{i},{T}_{n}\right)}{{H}_{1}\left({A}_{i},{T}_{n-1}\right)}\right)}^{l}={\left(\frac{{g}^{l\left({{A}_{i}}^{\mathrm{*}}\cdot \:{{\:T}_{n}}^{\mathrm{*}}\right)}}{{g}^{l\left({{A}_{i}}^{\mathrm{*}}\cdot \:{{\:T}_{n-1}}^{\mathrm{*}}\right)})}\right)}^{l}={g}^{l{{A}_{i}}^{\mathrm{*}}\left({{T}_{n}}^{\mathrm{*}}-{{T}_{n-1}}^{\mathrm{*}}\right)}\end{array}$$

Chanllenge: After $\:{\mathcal{A}}_{v}$ completes the key generation and update query in Phase 1, it submits two equal-length plaintext messages $\:{M}_{0}$ and $\:{M}_{1}$ to $\:{C}_{H}$. Subsequently, $\:{C}_{H}$ tosses a coin $\:\beta\:\in\:\left\{\mathrm{0,1}\right\}$, encrypts the message $\:{M}_{\beta\:}$ by challenging the access structure $\:{\gamma\:}^{\mathrm{*}}$, and sends the ciphertext to $\:{\mathcal{A}}_{v}$. The generated ciphertext is constructed as follows:

15 $$\:\begin{aligned}{C}_{0}&={{sec}_{i}}_{\beta\:}Z\nonumber\\{C}_{1}&={g}^{c}\nonumber\\ {C}_{1,i}&={X}^{{\lambda\:}_{i}}{U}_{\rho\:\left(i\right)}^{-{r}_{i}}\nonumber\\{C}_{2,i}&={g}^{{r}_{i}}\nonumber\\ {C}_{3,i}&={L}^{-{r}_{i}}\end{aligned}$$

The generated ciphertext set is $\:{CT}_{\beta\:}=\left\{{C}_{0},{C}_{1},{C}_{1,i},{C}_{2,i},{C}_{3,i}\right\}$.

Therefore:

16 $$\:\begin{array}{c}\left\{\begin{array}{c}{CT}_{\beta\:}=\left\{{{sec}_{i}}_{\beta\:}{e\left(g,g\right)}^{abc},{{C}_{1},C}_{1,i},{C}_{2,i},{C}_{3,i}\right\},\:\beta\:=0\\\:{CT}_{\beta\:}=\left\{{{sec}_{i}}_{\beta\:}{e\left(g,g\right)}^{z},{{C}_{1},C}_{1,i},{C}_{2,i},{C}_{3,i}\right\},\:\:\:\:\beta\:=1\end{array}\right.\end{array}$$

Let $\:s=c$, when $\:\beta\:=0$, $\:{C}_{0}={{sec}_{i}}_{\beta\:}{e\left(g,g\right)}^{abc}={{sec}_{i}}_{\beta\:}{Y}^{s}$, then $\:{CT}_{\beta\:}$ is a correct and legal ciphertext in our scheme.

Phase 2: The adversary $\:{\mathcal{A}}_{v}$ repeats the query operation of Phase 1, and the attribute set it queries does not satisfy the challenge access structure $\:{\gamma\:}^{\mathrm{*}}$.

Guess: The adversary $\:{\mathcal{A}}_{v}$ outputs a guess $\:{\beta\:}^{\mathrm{*}}$ for $\:\beta\:$. $\:{\mathcal{A}}_{v}$ wins the attack game only when $\:{\beta\:}^{\mathrm{*}}=\beta\:$. According to the above steps, define $\:{\mathcal{A}}_{v}$’s advantage in this game as:

17 $$\:\:\:\:\:\:\:\:\:\:\:\:\:\:\:\:\:\:\:\:\:\:\:\:\:\:\:\:\:\:\:Adv\left({\mathcal{A}}_{v}\right)=\left|{Pr}\left({\beta\:}^{*}=\beta\:\right)-\raisebox{1ex}{$1$}\!\left/\:\!\raisebox{-1ex}{$2$}\right.\right|\:$$

The following is divided into two situations for analysis:

When $\:\mu\:=1$, the ciphertext is random, $\:{G}_{2}$ cannot obtain information related to the plaintext, and its guess of $\:\beta\:$ is random, so:

18 $$\:\begin{array}{c}{Pr}\left({\beta\:}^{\mathrm{*}}=\beta\:|\mu\:=1\right)=\raisebox{1ex}{$1$}\!\left/\:\!\raisebox{-1ex}{$2$}\right.\end{array}$$

When $\:{\beta\:}^{\mathrm{*}}\ne\:\beta\:$, $\:\mathcal{B}$ outputs $\:{\mu\:}^{{\prime\:}}=1$, so:

19 $$\:\begin{array}{c}{Pr}\left({\mu\:}^{{\prime\:}}=\mu\:|\mu\:=1\right)=\raisebox{1ex}{$1$}\!\left/\:\!\raisebox{-1ex}{$2$}\right.\end{array}$$

When $\:\mu\:=0$, $\:{CT}_{\beta\:}$ is a legal ciphertext. Based on the above assumptions, $\:{\mathcal{A}}_{v}$’s advantage of breaking this scheme is ε, so:

20 $$\:\begin{array}{c}{Pr}\left({\beta\:}^{\mathrm{*}}=\beta\:|\mu\:=0\right)=\raisebox{1ex}{$1$}\!\left/\:\!\raisebox{-1ex}{$2$}\right.+\epsilon\:\end{array}$$

When $\:{\beta\:}^{\mathrm{*}}=\beta\:$, $\:\mathcal{B}$ outputs $\:{\mu\:}^{{\prime\:}}=0$, so:

21 $$\:\begin{array}{c}{Pr}\left({\mu\:}^{{\prime\:}}=\mu\:|\mu\:=0\right)=\raisebox{1ex}{$1$}\!\left/\:\!\raisebox{-1ex}{$2$}\right.+\epsilon\:\end{array}$$

From the above analysis, it can be seen that the advantage of $$\:\mathcal{B}$$ in solving the DBDH problem is $$\:\raisebox{1ex}{$1$}\!\left/\:\!\raisebox{-1ex}{$2$}\right.{Pr}\left({\mu\:}^{{\prime\:}}=\mu\:|\mu\:=0\right)+\raisebox{1ex}{$1$}\!\left/\:\!\raisebox{-1ex}{$2$}\right.{Pr}\left({\mu\:}^{{\prime\:}}=\mu\:|\mu\:=1\right)-\raisebox{1ex}{$1$}\!\left/\:\!\raisebox{-1ex}{$2$}\right.=\raisebox{1ex}{$\epsilon\:$}\!\left/\:\!\raisebox{-1ex}{$2$}\right.$$.

### Experimental analysis

We have used the cryptographic library JPBC (Java Pairing-Based Cryptography, a pairing-based Java cryptography library) on the IntelliJ IDEA 2022.3 software to implement our scheme, and uses the Type-a curve $\:{y}^{2}=\:{x}^{3}+\:x\:$ to handle the pairing operation. The experiments were conducted on a computer equipped with an *Intel Core i7* —12,700 K CPU @ 3.60 gHz, 32 GB RAM, and running the Windows 11 Professional operating system. In our scheme, the permission node $\:holds$ the chameleon hash sub-private key in the blockchain copyright transaction, and its withdrawal will affect the $\:redacting$ of copyright transaction data. This section performs performance analysis and testing on the exit time of permission nodes, and compares it with the solution in literature^69–73^. The exit time of a permission node refers to the time between the start time when a node issues an exit request and the time when other permission nodes agree to exit. Assume that the exit request time issued by the exit node is $\:ExitRequest$, the node agrees to exit time is $\:Withdraw$, and the permission node exit time can be expressed as $\:ExitTime=Withdraw-ExitRequest$, which includes three stages of encrypted broadcast, decryption and verification.

Set the total number of permission nodes to $\:n$ $\:(20\le\:n\le\:120),\:the\:node\:applying\:for\:exit\:is\:{x}_{i},\:the\:number\:of\:other\:permission\:nodes\:is\:\left(n-1\right),\:$the exit node will provide its sub-private key to the other $\:\left(n-1\right)$ nodes. In order to ensure confidentiality during the broadcast of the sub-private key, the exit node uses the KEA-CPABE-UK algorithm to encrypt the broadcast of the sub-private key.

**Fig. 7:**
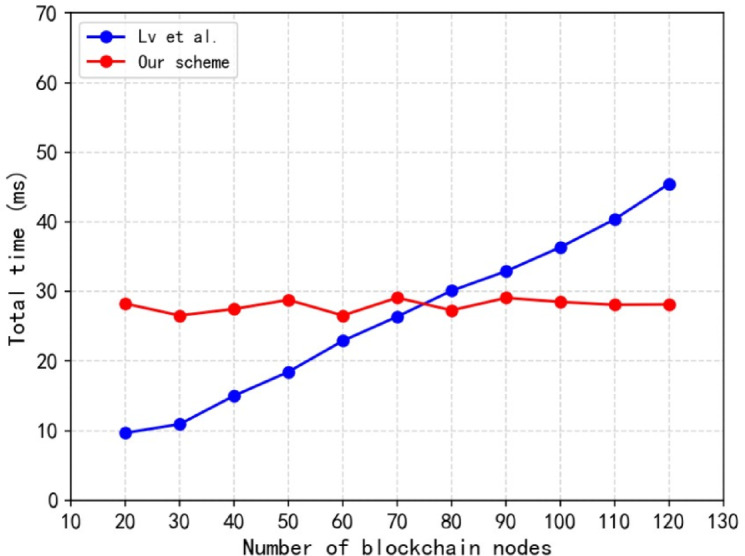
Comparison of encrypted broadcast time of permission nodes.

Based on $\:{Z}_{p}\left(\left|p\right|=80\right)$, $\:{G}_{q}\left(\left|q\right|=256\right)$ parameter space, the broadcast time of the scheme proposed by Lv et al.^74^ and our scheme under different numbers of blockchain permission nodes is shown in Fig. 7. The access structure specifies that the node must satisfy the given attributes to be decrypted correctly. Let the number of access attributes be 6 and the total number of attributes be 6. It can be seen from the experimental results that when the number of permission nodes $\:n<75$, the sub-private key broadcasting time of our scheme is higher than that of Lv et al.^74^. When $\:n>75$, the sub-private key broadcast of this scheme has smaller time overhead. Therefore, in a multi- permission node scenario, the sub-private key broadcasting efficiency of this solution is higher, and it is suitable for the large-scale copyright blockchain when the number of permission nodes is large.

As the number of permission node increases, the sub-private key broadcast time of the scheme proposed by Lv et al.^74^ increases linearly, while the sub-private key broadcast time of this scheme tends to be stable. This is because the KEA-CPABE-UK algorithm used in this solution only needs to encrypt the sub-private key through public parameters, and other permission nodes can complete the decryption, which does not depend on the number of permission nodes. The solution of Lv et al.^74^ adopts the public key cryptography system that comes with the traditional blockchain. The one-to-one encryption mode makes its encryption broadcast time grow linearly with the increase of permission nodes in the blockchain.

**Fig. 8:**
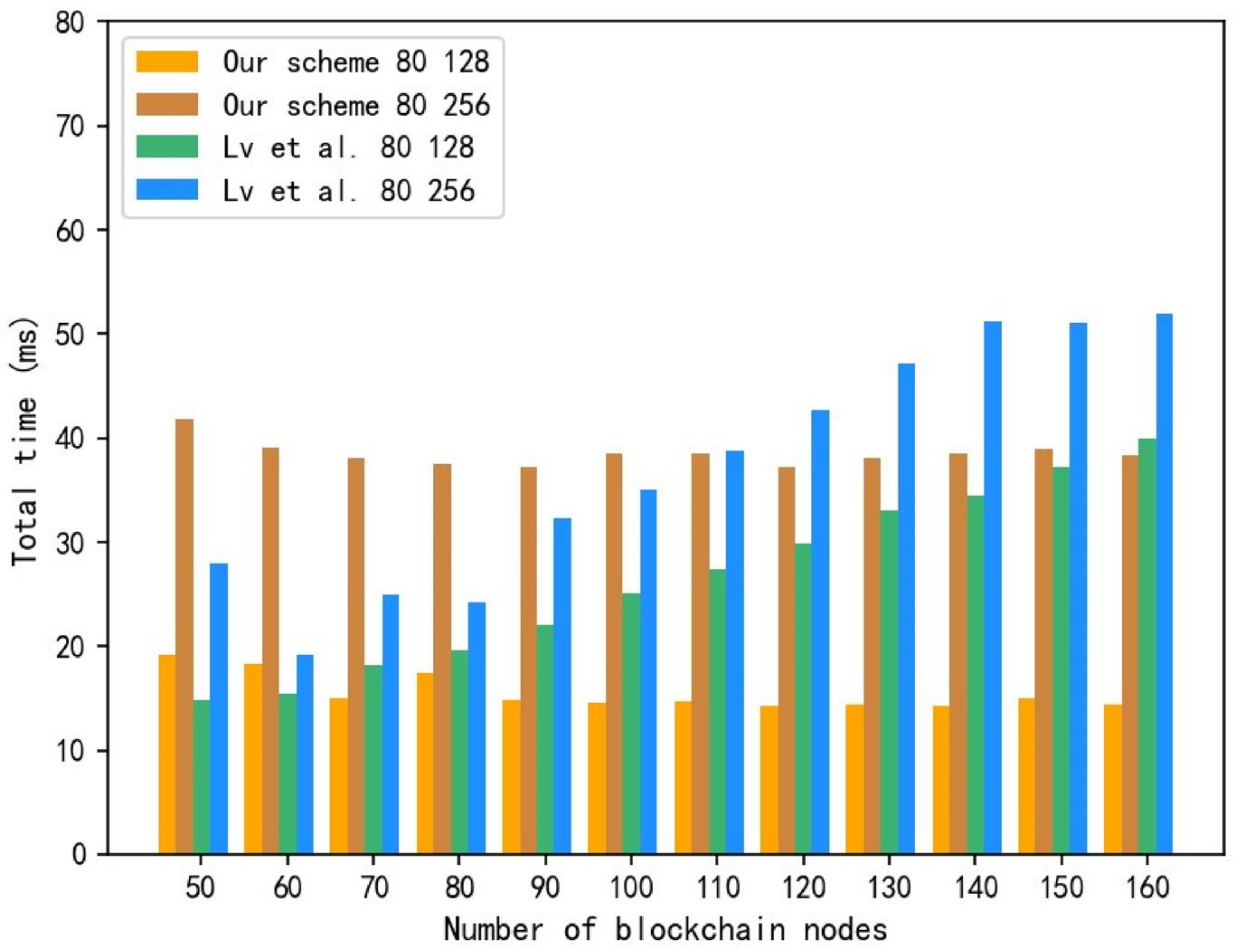
Comparison of exit time of permission nodes.

The node exit time $\:ExitTime$ includes three stages: encrypted broadcast, decryption and verification. After other permission nodes obtain the plaintext of the sub-private key of the exit node, they execute the $\:Verify$ algorithm to verify the correctness of the sub-private key. Since the verification process remains unchanged, the $\:ExitTime$ involved in the experiment mainly focuses on the encryption broadcast and decryption stages. The relationship between the number of blockchain permission nodes and the exit time of permission nodes in our scheme and Lv et al.^74^ scheme under different parameter spaces are shown in Fig. 8.

When the parameter spaces are different, the total time required for permission node exit in our scheme and Lv et al.^74^ scheme increases as the space increases. Under the same parameter space, the node exit time of this scheme is significantly lower than the scheme of Lv et al.^74^ when the number of permission nodes gradually increases $\:\left(n\ge\:120\right)$. This is because when the number of nodes reaches a certain number, the independence of the KEA-CPABE-UK algorithm prevents the solution time consumption from increasing linearly with the increase in the number of nodes. Compared with the scheme of Lv et al.^74^, our scheme has obvious advantages in the time cost spent in the permission node exit phase. Therefore, the total exit time of this scheme does not depend on the number of nodes. When the parameter space is certain, the exit time of permission nodes tends to be stable.

**Fig. 9:**
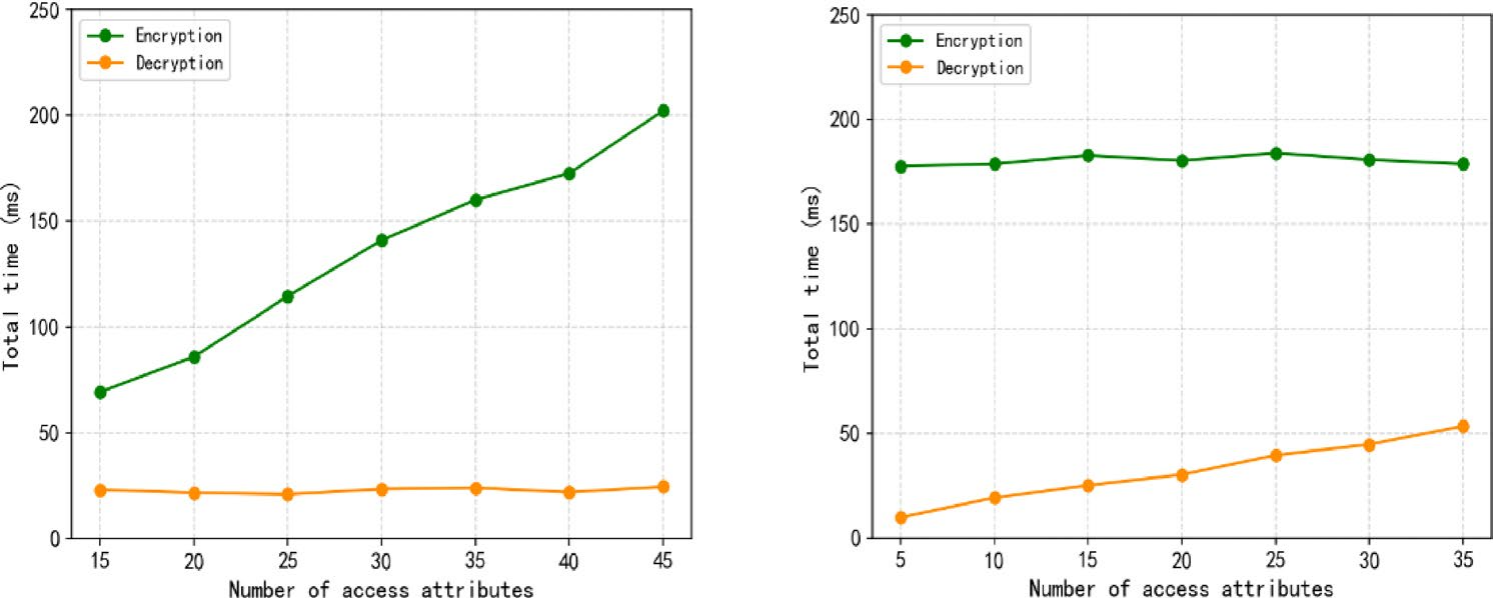
Encryption and decryption time overhead (**a**) The number of total attributes is different (**b**) The number of accessed attributes is different.

In order to compare the actual running time cost of encryption and decryption of node sub-private keys under different access structures, we simulated the encryption and decryption time cost of permission nodes in two situations and conducted experimental tests. The first is the encryption and decryption time overhead of the permission node when the number of accessed attributes is fixed and the total number of attributes is different. The second is the encryption and decryption time overhead when the total number of attributes is constant and the number of accessed attributes is different. Among them, the access structure specifies that the node must satisfy a given number of attributes to be decrypted correctly. The experimental running results are shown in Table 3. Figure 9 visually compares the encryption and decryption time overhead of nodes in the two cases.

**Table 3:** Node encryption and decryption running time costs under different circumstances.

Access structure	Size	Encryption time	Decryption time
The running time when the access attributes are fixed but the total attributes are different			
Access attributes = 15	Total attributes = 15	177.67ms	9.83 ms
	Total attributes = 20	178.75 ms	19.17 ms
	Total attributes = 25	182.83 ms	25.17 ms
	Total attributes = 30	180.33 ms	30.25 ms
	Total attributes = 35	184.00 ms	39.58 ms
	Total attributes = 40	180.75 ms	44.75 ms
	Total attributes = 45	178.75 ms	53.50 ms
The running time when the total attributes are fixed and the access attributes are different			
Total attributes = 40	Access attributes = 5	69.33 ms	23.0 ms
	Access attributes = 10	86.00 ms	21.67 ms
	Access attributes = 15	114.50 ms	20.92 ms
	Access attributes = 20	140.92 ms	23.42 ms
	Access attributes = 25	160.08 ms	24.00 ms
	Access attributes = 30	172.67 ms	21.92 ms
	Access attributes = 35	202.25 ms	24.50 ms

As shown in Fig. 9a, let the total number of attributes be 15–45. When the number of accessed attributes is 15 and remains unchanged, the node encryption time increases as the total number of attributes increases, while the decryption time tends to stabilize. This is because node encryption requires more attributes and the time cost is higher, while the decryption time is only related to the number of accessed attributes. When the total number of attributes is constant, the more the number of accessed attributes, the longer the decryption of the node takes, and its encryption broadcast time is not affected by the number of accessed attributes, as shown in Fig. 9 (b). Therefore, the appropriate number of attributes and access structure can have obvious advantages when the number of permission nodes is large.

#### Discussion on Trade-offs

Our scheme has introduced a marginal increase in computational latency during the threshold key reconstruction phase compared to Lv et al. However, this is a necessary trade-off to eliminate the centralized trust risk, thereby significantly enhancing the security and decentralization of the IoT system.

### Performance analysis in IoT scenarios

To further validate the practical deployment potential of the proposed scheme in resource-constrained IoT environments, we have conducted comprehensive simulation experiments focusing on two dimensions: transaction latency and storage sustainability.

#### Transaction latency and scalability

Transaction latency is a pivotal metric for evaluating whether a blockchain system can support real-time copyright services in IoT networks. We have measured the end-to-end latency, which includes attribute encryption, signature generation, consensus propagation, and chameleon hash-based verification, across a varying number of permission nodes (from 4 to 40).

**Fig. 10:**
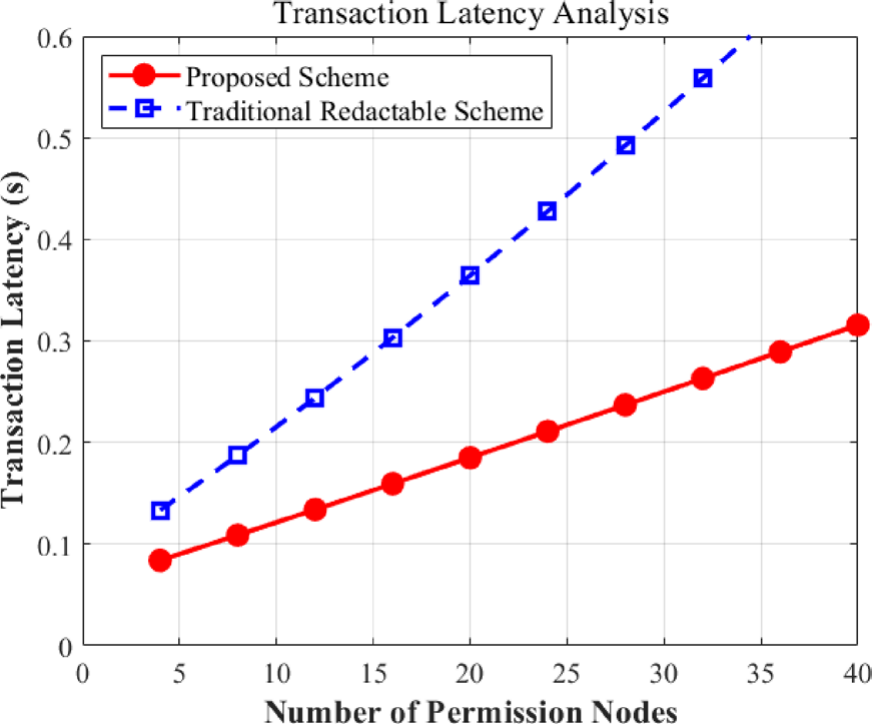
Transaction latency under varying permission node scales.

As illustrated in Fig. 10, the transaction latency of the proposed scheme has exhibited a stable and sub-linear growth trend. Specifically, when the network scale is small (e.g., 4 nodes), the latency is approximately 0.12s. As the scale expands to a relatively large IoT consensus group of 40 nodes, the latency only increases to 0.35s. In contrast, traditional redactable blockchain schemes (e.g., those relying on heavy secret sharing or chain restructuring) show a sharper increase, exceeding 0.55s at the 40-node mark.

The superior performance of our scheme stems from the multi-center architecture, which parallelizes the attribute verification workload, and the efficient chameleon hash operation that avoids complex re-encryption during data redaction. This result has indicated that our framework is highly scalable and maintains acceptable response times even as the IoT network grows.

#### Storage efficiency under repeated redactions

In IoT-based copyright systems, frequent error corrections or ownership updates (redactions) can lead to a “storage explosion” if not handled properly. We have compared the cumulative storage overhead of our scheme against traditional redundancy-based methods over 20 consecutive redaction cycles.

**Fig. 11:**
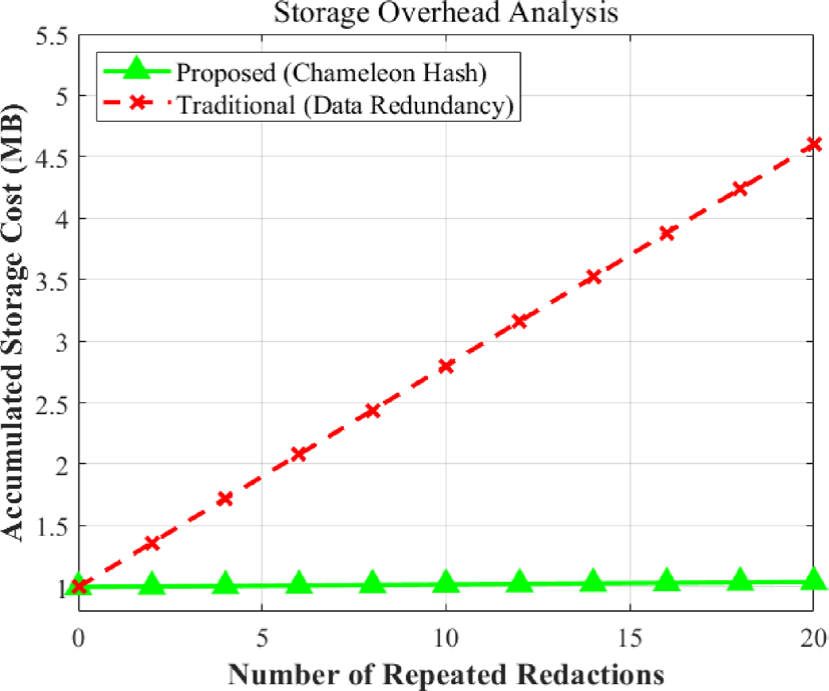
Cumulative storage overhead across repeated redaction cycles.

The experimental results are shown in Fig. 11, these have revealed a stark contrast. Traditional schemes incur a significant linear increase in storage, with the total data volume growing from an initial 1.0 MB to over 4.5 MB after 20 redactions. This is because traditional methods often retain all historical versions or create new blocks for every modification.

Conversely, the storage overhead of our proposed scheme remains nearly constant, hovering around 1.04 MB even after 20 redactions. The marginal increase (less than 4%) is solely due to the storage of minimal accountability metadata (e.g., redactor’s digital signature). By leveraging the “collision” property of chameleon hashing, our scheme achieves “in-place” updates without altering the blockchain’s block structure or adding redundant data. This characteristic is particularly crucial for IoT edge devices with limited memory, ensuring long-term operational sustainability without frequent data pruning.

## Conclusions

In this work we have analyzed the state of the art of the copyright transaction methods and the shortcomings of research on copyright transactions based on blockchain. In terms of copyright use, a blockchain based copyright transaction model and scheme that supports data editability is proposed. In response to the data redactionredacting requirements brought about by ownership changes, the data redacting method based on chameleon hash is used to complete the modification of copyright registration information in the transaction process. In order to prevent permission nodes from doing evil, a flexible and controllable permission node selection, exit and change mechanism is designed. The KEA-CPABE-UK algorithm is introduced to provide security protection for chameleon hash sub-private keys. Experimental results and security analysis have shown that our scheme has satisfied the confidentiality of the copyright transferor’s private information, resistance to malicious node operations, and the confidentiality and accountability of the chameleon hash private key. In scenarios with many permission nodes, the sub-private key broadcast during the node exit phase has higher efficiency and flexibility. So, the proposed method is proven to be very effective for data confidentiality of the copyright transferor, resistance to malicious nodes, and the confidentiality and accountability of the private key of the chameleon hash in the BIoT and BIIoT systems.

In future work, we will focus on optimizing the proposed scheme for resource-constrained IoT devices. Specifically, we have a plan to implement lightweight cryptographic primitives, such as replacing standard elliptic curves with more efficient Edwards-curve Digital Signature Algorithm (EdDSA) or utilizing hardware acceleration. Additionally, we will explore edge computing offloading strategies to delegate the heavy computational tasks of attribute-based encryption (CP-ABE) from IoT terminals to edge gateways, further reducing the local energy consumption and processing latency.

## Data Availability

All data are in the paper.

## References

[1] Nakamoto, S. Bitcoin: A peer-to-peer electronic cash system, [Online] (2008). Available: https://bitcoin.org/bitcoin.pdf, [Accessed: Mar. 3, 2026].

[2] Monostori, L. et al. Cyber-physical systems in manufacturing. *CIRP Ann.* **65** (2), 621–641. 10.1016/j.cirp.2016.06.005 (2016).

[3] Li, Z., Barenji, A. V. & Huang, G. Q. Toward a Blockchain cloud manufacturing system as a peer to peer distributed network platform. *Robot Comput. Integr. Manuf.* **54**, 133–144. 10.1016/j.rcim.2018.05.011 (2018).

[4] Yu, T., Lin, Z. & Tang, Q. Blockchain: The introduction and its application in financial accounting. *J. Corp. Acc. Finance*. **29** (4), 37–47. 10.1002/jcaf.22365 (2018).

[5] Vora, J. et al. Bheem: A Blockchain-based framework for securing electronic health records. In *2018 IEEE Globecom Workshops (GC Wkshps)*. 1–6. 10.1109/GLOCOMW.2018.8644088 (2018).

[6] Sethi, A. & Sethi, S. Flexibility in manufacturing: A survey. *Int. J. Flex. Manuf. Syst.* **2** (4). 10.1007/BF00186471 (1990).

[7] Lee, J., Kao, H. A. & Yang, S. Service innovation and smart analytics for Industry 4.0 and big data environment. *Procedia CIRP*. **16**, 3–8. 10.1016/j.procir.2014.02.001 (2014).

[8] Lee, J., Bagheri, B. & Kao, H. A. A Cyber-Physical Systems architecture for Industry 4.0-based manufacturing systems. *Manuf. Lett.* **3**, 18–23. 10.1016/j.mfglet.2014.12.001 (2015).

[9] Yang, L. Industry 4.0: A survey on technologies, applications and open research issues. *J. Ind. Inf. Integr.* **6**, 1–10. 10.1016/j.jii.2017.04.005 (2017).

[10] Xu, X. From cloud computing to cloud manufacturing. *Robot Comput. Integr. Manuf.* **28**, 75–86. 10.1016/j.rcim.2011.07.002 (2012).

[11] Palma, L. M. et al. Blockchain and smart contracts for higher education registry in brazil. *Int. J. Netw. Manag.* **29**(3), e2061. 10.1002/nem.2061 (2019).

[12] Zissis, D. & Lekkas, D. Addressing cloud computing security issues. *Futur Gener Comput. Syst.* **28** (3), 583–592. 10.1016/j.future.2010.12.006 (2012).

[13] Swan, M. Rezension Blockchain: Blueprint for a New Economy. *HMD* **55**, 1362–1364. 10.1365/s40702-018-00468-4 (2015).

[14] Bhattacharjya, A., Zhong, X., Wang, J. & Xing, L. Security Challenges and Concerns of Internet of Things (IoT), In: (eds Guo, S. & Zeng, D.) Cyber-Physical Systems: Architecture, Security and Application, EAI/Springer Innovations in Communication and Computing, Springer, Cham: 153–185.

[15] Bhattacharjya, A., Zhong, X., Wang, J. & Xing, L. Secure IoT Structural design for Smart Cities, In Smart Cities Cybersecurity and Privacy, Elsevier: 187–201.

[16] Bhattacharjya, A., Zhong, X., Wang, J. & Xing, L. Present Scenarios of IoT Projects with Security Aspects Focused, In: (eds Farsi, M., Daneshkhah, A., Hosseinian-Far, A. & Jahankhani, H.) Digital Twin Technologies and Smart Cities. Internet of Things (Technology, Communications and Computing), Springer, Cham: 95–122, 10.1007/978-3-030-18732-3_7.

[17] Bhattacharjya, A., Zhong, X., Wang, J. & Xing, L. CoAP—Application Layer Connection-Less Lightweight Protocol for the Internet of Things (IoT) and CoAP-IPSEC Security with DTLS Supporting CoAP, In: (eds Farsi, M., Daneshkhah, A. & Hosseinian-Far, A.) Jahankhani H. Digital Twin Technologies and Smart Cities. Internet of Things (Technology, Communications and Computing), Springer, Cham: 151–175, 10.1007/978-3-030-18732-3_9.

[18] IBM Blockchain based on Hyperledger Fabric from the Linux Foundation. [Online]. Available:. (2017). https://www.ibm.com/Blockchain/hyperledger. [Accessed: Feb. 9, 2025].

[19] Developer Hub, I. O. T. A. [Online]. (2017). Available: https://www.iota.org/research/meetthe-tangle. [Accessed: Feb. 9, 2026].

[20] Pustišek, M. & Kos, A. Approaches to Front-End IoT Application Development for the Ethereum Blockchain. *Procedia Comput. Sci. vol*. **129**, 410–419. 10.1016/j.procs.2018.03.017 (2018).

[21] Viktor Trón, F. L. & Ethereum Specification [Online]. (2015). Available: https://github.com/ethereum/go-ethereum/wiki/Ethereum-Specification. [Accessed: Feb. 9, 2025].

[22] Kim, M. et al. Blockchain-Enabled Maximum Evacuation System Using Hybrid Voting in Zero Trust Hiking Trail and Mountainous Terrain, *IEEE Internet Things J.*, **12**, 5, 5847–5858, 10.1109/JIOT.2024.3490560. (2025).

[23] Crosby, M. et al. Blockchain technology: Beyond bitcoin, *Applied Innovation*, no.2, pp.(6–10). [Online]. (2016). Available: https://scet.berkeley.edu/wp-content/uploads/AIR-2016-Blockchain.pdf. [Accessed: Feb. 9, 2026].

[24] Bailis, P. et al. Research for practice: cryptocurrencies, Blockchains, and smart contracts; hardware for deep learning. *Commun. ACM*. **60** (5), 48–51. 10.1145/3024928 (2017).

[25] Aste, T., Tasca, P. & Centre, U. C. L. Blockchain technologies: The foreseeable impact on society and industry. *Computer ***50**(9), 18–28. 10.1109/MC.2017.3571064 (2017).

[26] Cachin, C. & Vukoli, M. Blockchains Consensus Protocols in the Wild, *arXiv preprint* arXiv:1707.01873. 10.48550/arXiv.1707.01873 (2017).

[27] Iota. a cryptocurrency for Internet-of-Things. url: https://iota.org/.

[28] Tangle. url: https://iota.org/IOTA_Whitepaper.pdf.

[29] Bano, S. et al. Consensus in the Age of Blockchains. In *Proceedings of the 1st ACM Conference on Advances in Financial Technologies*, pp. 183–198. 10.1145/3318041.3355458 (2019).

[30] Wang, W. et al. A survey on consensus mechanisms and mining strategy management in Blockchain networks. *IEEE Access.* **7**, 22328–22370. 10.1109/ACCESS.2019.2896108 (2019).

[31] Banerjee, M., Lee, J. & Raymond Choo, K. K. A Blockchain future for internet of things security: a position paper. *Digit. Commun. Networks*. **4** (3), 149–160. 10.1016/j.dcan.2017.10.006 (2017).

[32] Baliga, A. Understanding Blockchain Consensus Models. April, [Online]. (2017). Available: https://www.persistent.com/wp-content/uploads/2017/04/WP-Understanding-Blockchain-Consensus-Models.pdf. [Accessed: Feb. 9, 2025].

[33] Pilkington, M. Blockchain technology: principles and applications, *Research handbook on digital transformations*, pp. 225–253. 10.4337/9781784717766.00019 (2016).

[34] Sankar, L. S., Sindhu, M. & Sethumadhavan, M. Survey of consensus protocols on Blockchain applications, In *4th international conference on advanced computing and communication systems (ICACCS)*. IEEE, pp.1–5. 10.1109/ICACCS.2017.8014672 (2017).

[35] Underwood, S. Blockchain beyond bitcoin. *Commun. ACM*. **59** (11), 15–17. 10.1145/2994581 (2016).

[36] Seibold Sigrid and Samman, George Consensus:Immutable agreement for the Internet of value, KPMG. [Online]. (2016). Available: https://assets.kpmg.com/content/dam/kpmg/pdf/2016/06/kpmgBlockchain-consensus-mechanism.pdf. [Accessed: Feb. 9, 2026].

[37] Mukhopadhyay, U. et al. A brief survey of cryptocurrency systems, In *Proceedings of the 14th annual conference on privacy, security and trust (PST)*. IEEE, pp. 745–752, (2016). 10.1109/PST.2016.7906988.

[38] Awasthi, D. et al. A comprehensive review on optimization-based image watermarking techniques for copyright protection. *Expert Syst. Appl.* **242** (122830). 10.1016/j.eswa.2023.122830 (2024).

[39] Chung, T. Y. et al. Digital watermarking for copyright protection of MPEG2 compressed video. *IEEE Trans. Consum. Electron.* **44** (3), pp895–901. 10.1109/30.713211 (1998).

[40] Bhattacharjya, A., Zhong, X. & Wang, J. Strong, efficient and reliable personal messaging peer to peer architecture based on hybrid RSA. In *Proceedings of The International Conference on Internet of Things and Cloud Computing (ICC*), pp. 1–5. 10.1145/2896387.2896431 (2016).

[41] Bhattacharjya, A. et al. An end-to-end user two-way authenticated double encrypted messaging scheme based on hybrid RSA for the future internet architectures. *International J. Inform. Comput. Security*. **10**, 63–79. 10.1504/IJICS.2018.089593 (2018).

[42] Bhattacharjya, A. & Zhong, X. al.Hybrid RSA-based highly efficient, reliable and strong personal full mesh networked messaging scheme. *Int. J. Inf. Comput. Secur.* **10** (4), 418–436. 10.1504/IJICS.2018.095341 (2018).

[43] Bhattacharjya, A. et al. On mapping of address and port using translation. *Int. J. Inf. Comput. Secur.* **11**(3), 214–232. 10.1504/IJICS.2019.099419 (2019).

[44] Feng, L. I. U., Jie, Y. A. N. G. & Jiayin, Q. I. Survey on blockchain privacy protection techniques in cryptography. *Chin. J. Netw. Inform. Secur.* **8** (4), 29–44. 10.11959/j.issn.2096-109x.2022054 (2022).

[45] Marinov, M., Kalmukov, Y. & Valova, I. Content-Based Image Retrieval: Impact of image resolution on the search accuracy and results ordering. In *International Conference Automatics and Informatics (ICAI)*, 2021, pp. 72–75. 10.1109/ICAI52893.2021.9639858 (2021).

[46] Zhang, Q. et al. Digital image copyright protection method based on blockchain and zero trust mechanism. *Multimedia Tools Appl.* **83**, 77267–77302. 10.1007/s11042-024-18514-3 (2024).

[47] T B, R S, K B, et al. Proxy re-encryption approach to avoid illegal content sharing in cloud. In *6th International Conference on Intelligent Computing and Control* Systems *(ICICCS)*, 2022, pp. 618–623. 10.1109/ICICCS53718.2022.9788263 (2022).

[48] Lee, K. et al. The Design of a DRM System Using PKI and a Licensing Agent. *Netw. Parallel Comput.* 611–617. 10.1007/978-3-540-30141-790 (2004).

[49] Tirkel, A. Z. et al. Electronic watermark. In *Digital Image Computing, Technology and Applications (DICTA’93)*, pp. 666–673. (1993).

[50] Hemdan, E. E. D. An efficient and robust watermarking approach based on single value decompression, multilevel DWT, and wavelet fusion with scrambled medical images. *Multimedia Tools Appl.* **80** (2), 1749–1777. 10.1007/s11042-020-09769-7 (2021).

[51] Xiang, S. J. & Yang, L. Robust and reversible image watermarking algorithm in homomorphic encrypted domain. *J. Softw.* **29** (4), 957–972. 10.13328/j.cnki.jos.005406 (2018).

[52] Kadian, P., Arora, S. M. & Arora, N. Robust Digital Watermarking Techniques for Copyright Protection of Digital Data: A Survey. *Wireless Pers. Commun.* **118** (4), 3225–3249. 10.1007/s11277-021-08177-w (2021).

[53] Shen, M. et al. Content-based multi-source encrypted image retrieval in clouds with privacy preservation. *Future Generation Comput. Syst.* **109**, 621–632. 10.1016/j.future.2018.04.089 (2020).

[54] Yingying, L. I., Jianfeng, M. A. & Yinbin, M. I. A. O. Encrypted image retrieval in multi-key settings based on edge computing. *J. Commun.* **41** (4), 14–26. 10.11959/j.issn.1000-436x.2020086 (2020).

[55] Guo, J. et al. Blockchain-enabled digital rights management for multimedia resources of online education. *Multimedia Tools Appl.* **79**, 9735–9755. 10.1007/s11042-019-08059-1 (2020).

[56] Ku, W. & Chi, C. H. Survey on the technological aspects of digital rights management. In *Information Security: 7th International Conference*, pp. 391–403. (2004). 10.1007/978-3-540-30144-8_33.

[57] Subramanya, S. R. & Yi, B. K. Digital rights management. *IEEE Potentials*. **25** (2), 31–34. 10.1109/mp.2006.1649008 (2006).

[58] Zhu, P. et al. Using blockchain technology to enhance the traceability of original achievements. *IEEE Trans. Eng. Manage.* 1–15. 10.1109/TEM.2021.3066090 (2021).

[59] Yang, Y. et al. A Video Copyright Transaction Traceability Method Based on Mother-Child Blockchain. In *the 3rd International Conference on Blockchain Technology and Applications*, pp. 1–6, 2021. 10.1145/3446983.3446984 (2020).

[60] Bachani, V., Wan, Y. & Bhattacharjya, A. Preferential DPoS: a scalable blockchain schema for high-freuency transaction. = *AMCIS 2022 TREOs*. 36. https://aisel.aisnet.org/treos_amcis2022/36.

[61] Gu, H., Shang, J., Wang, P., Mi, J. & Bhattacharjya, A. A secure protocol authentication method based on the strand space model for blockchain-based industrial internet of things. *Symmetry-Basel ***16**(7), 851. 10.3390/sym16070851 (2024).

[62] Kumar, J. R. et al. Blockchain based traceability in computer peripherals in universities scenarios. In *3rd International Conference on Electronic and Electrical Engineering and Intelligent System (ICE3IS)*. IEEE, 2023. 10.1109/ICE3IS59323.2023.10335420 (2023).

[63] Si, P. et al. Distributed sender scheduling for multimedia transmission in wireless mobile peer to-peer networks. *IEEE Trans. Wireless Commun.* **8**, 4594–4603. 10.1109/twc.2009.080550 (2009).

[64] Huang, J. et al. Towards Secure Industrial IoT: Blockchain System With Credit-Based Consensus Mechanism. *IEEE Trans. Industr. Inf.* **15**, 3680–3689. 10.1109/TII.2019.2903342 (2019).

[65] Zheng, Z. et al. An overview on smart contracts: Challenges, advances and platforms. *Future Generation Comput. Syst.* **105**, 475–491. 10.1016/j.future.2019.12.019 (2020).

[66] Zou, W. et al. Smart Contract Development: Challenges and Opportunities. *IEEE Trans. Software Eng.* **47** (10), 2084–2106. 10.1109/TSE.2019.2942301 (2021).

[67] Liang, W. et al. A dual-chain digital copyright registration and transaction system based on blockchain technology. *Blockchain Trustworthy Syst.* 702–714. 10.1007/978-981-15-2777-7_57 (2021).

[68] Zhang, C. et al. A trustworthy industrial data management scheme based on redactable blockchain. *J. Parallel Distrib. Comput.* **vol,152**, 167–176. 10.1016/j.jpdc.2021.02.026 (2021).

[69] Huang, K. et al. Building Redactable Consortium Blockchain for Industrial Internet-of Things. *IEEE Trans. Industr. Inf.* **15** (6), 3670–3679. 10.1109/TII.2019.2901011 (2019).

[70] Ateniese, G. et al. Redactable Blockchain – or – Rewriting History in Bitcoin and Friends, in *2017 IEEE European Symposium on Security and Privacy (EuroS&P)*, pp.111–126, [Online]. (2017). Available: 10.1109/EuroSP.2017.37 [Accessed: Feb. 9, 2025].

[71] LI, P. L., XU H X, MA T J, MU & Y H. Research on Fault-correcting Blockchain Technology. *J. Cryptologic Res.* **5** (5), 501–509. 10.13868/j.cnki.jcr.000259 (2018).

[72] Fan, S. & Chen, Y. Editable Blockchain Scheme Based on Shamir Chameleon Hash Secret Sharing. In *IEEE 6th Information Technology and Mechatronics Engineering Conference (ITOEC)*, vol.6, pp.1125–1128. 10.1109/ITOEC53115.2022.9734554 (2022).

[73] Xiaoqi, Z. H. A. O., Zhenghao, Z. H. A. N. G. & Yong, L. I. An Editable and Accountable Blockchain Scheme. *J. Cyber Secur.* **7** (5), 19–28. 10.19363/J.cnki.cn10-1380/tn.2022.09.02 (2022).

[74] Wei-Long, L. V. et al. Research on Verifiable Blockchain Ledger Redaction Method for Trusted Consortium. *Chin. J. Comput.* **44** (10), 2016–2032. 10.11897/SP.J.1016.2021.0201 (2021).

